# Early malaria risk screening in Nigerian minors using AutoML and cluster-based analysis of non-clinical survey data

**DOI:** 10.1371/journal.pdig.0001736

**Published:** 2026-09-23

**Authors:** Ashiqur Rahman Khan, Nafisa Mahbub, Ridwan Al Aziz, Mahamudul Hassan Siddique, Zahidul Islam Sayeem

**Affiliations:** Department of Industrial and Production Engineering, Bangladesh University of Engineering and Technology (BUET), Dhaka, Bangladesh; Instituto Politécnico Nacional Escuela Superior de Medicina: Instituto Politecnico Nacional Escuela Superior de Medicina, MEXICO

## Abstract

Malaria remains a major public health challenge in sub-Saharan Africa, with Nigeria accounting for a substantial proportion of the global malaria burden, particularly among children under five years of age. Although rapid diagnostic tests (RDTs) enable timely screening, false-negative results and delays in confirmatory microscopy can hinder early treatment in resource-limited settings. This study developed and evaluated an automated machine learning (AutoML)-based framework for early malaria risk prediction using demographic, household, and socioeconomic data from the nationally representative Nigeria Malaria Indicator Survey (MIS) 2021. Initially, 43 candidate variables were selected and subsequently reduced to 13 statistically significant predictors through preprocessing and statistical dependency testing. Five machine learning models, namely Logistic Regression (LR), Decision Tree (DT), Support Vector Classifier (SVC), Extreme Gradient Boosting (XGBoost), and H2O AutoML, were developed and evaluated using stratified train–test splitting and 10-fold cross-validation. To reduce the influence of regional information, Agglomerative Hierarchical Clustering (AHC) was performed using the eight most important predictors after excluding regional identifiers, yielding two optimal population subgroups (Silhouette Score = 0.3939). For the overall dataset, H2O AutoML Generalized Linear Model (GLM) achieved the highest F_2_-score (79.38%) with a recall of 83.08%, whereas XGBoost achieved the highest test accuracy (75.54%). Cluster-specific analyses demonstrated that H2O GLM provided the best recall-oriented performance in Cluster 1 (F_2_-score = 83.31%), while XGBoost performed best in Cluster 2 (F_2_-score = 83.25%). These findings demonstrate that H2O AutoML provides a robust and automated framework for malaria risk prediction, while cluster-specific modeling further improves predictive performance by accounting for population heterogeneity. The proposed framework has the potential to support early malaria risk assessment and complement conventional diagnostic strategies in resource-limited public health settings.

## Author summary

Effective malaria control depends on the timely identification of children at risk so that appropriate treatment can be initiated before severe disease develops. In Nigeria, rapid diagnostic tests may produce false-negative results, while delays in confirmatory laboratory testing can hinder timely clinical decision-making, particularly in resource-limited settings. In this study, we developed an automated machine learning framework using routinely available demographic, household, and socioeconomic information from the 2021 Nigeria Malaria Indicator Survey to support early malaria risk prediction. To reduce the influence of regional information on model development, agglomerative hierarchical clustering was performed after excluding regional identifiers, enabling the identification of population subgroups with distinct risk profiles. H2O AutoML demonstrated strong recall-oriented performance and produced competitive predictive models across both the overall population and cluster-specific analyses. These findings suggest that combining non-clinical data, automated machine learning, and subgroup-specific modeling can support scalable and more targeted malaria risk assessment, complementing existing diagnostic strategies in resource-limited settings.

## Introduction

Malaria is a life-threatening infectious disease caused by *Plasmodium* parasites and transmitted primarily through the bites of infected female *Anopheles* mosquitoes. Among the five species that infect humans, *Plasmodium falciparum* poses the greatest threat in sub-Saharan Africa, where it is responsible for the majority of severe cases and fatalities. Despite being preventable and treatable, malaria remains a major public health burden: in 2021 alone, an estimated 247 million cases and 619,000 deaths were reported globally, with the African region accounting for approximately 95% of cases and 96% of deaths. Nigeria bears the highest burden, contributing more than 31% of global malaria-related deaths, disproportionately affecting children under five, pregnant women, and immunocompromised populations [1].

Early diagnosis is critical, as untreated *P. falciparum* infections can rapidly progress to severe illness or death within 24 hours. Although rapid diagnostic tests (RDTs) provide results within minutes, they are prone to false negatives [2], particularly in early-stage or low-parasitemia infections. In such cases, confirmatory microscopy using repeated blood smears over several days is required, delaying treatment initiation and increasing mortality risk [3]. Evidence from the dataset used in this study further highlights this challenge, where a substantial number of initially negative RDT results were later confirmed as positive through blood smear tests [4].

Recent advances in machine learning offer opportunities to complement conventional diagnostics by enabling early risk screening using non-clinical information, such as household, demographic, and socio-economic variables that are readily available in large-scale surveys. However, existing studies often rely on manually tuned models, clinical features, or geographically biased predictors, limiting scalability and equity. To address these gaps, this study leverages Automated Machine Learning (AutoML) [5] to develop robust, recall-oriented malaria risk models while minimizing human bias in model selection and tuning. Furthermore, clustering-based stratification is employed to reduce spatial-bias and tailor predictions to heterogeneous population subgroups, promoting more equitable and scalable early screening in resource-constrained settings.

Decision-making technologies such as machine learning (ML) and deep learning (DL) have opened a new era in the healthcare sector by enabling rapid analysis of patient histories using multiple parameters simultaneously. In clinical settings, timely decision-making is critical, as even a fraction of a second can be vital for patient outcomes. The integration of ML/DL techniques helps reduce human error and enhances the efficiency and accuracy of disease prediction. In the case of malaria, early diagnosis remains challenging because the disease is often difficult to detect before clear symptoms appear, and *rapid diagnostic tests* may sometimes yield inaccurate results. To address these challenges, researchers have increasingly applied predictive models using diverse data sources, including clinical factors, RNA sequencing data, medical images from blood samples, biological indicators, meteorological variables, and socio-economic information. In recent years (2022–2025), substantial progress has been achieved in malaria prediction through advances in ML and DL methodologies, supported by improved data availability and multimodal data integration. Consequently, contemporary studies demonstrate enhanced diagnostic accuracy and improved capabilities for malaria detection and outcome prediction.

Several studies have focused on malaria diagnosis using clinical and epidemiological data. Sekhar and Alemu [6] conducted a comprehensive comparative analysis of ML models using a large, rigorously validated synthetic clinical dataset representing sub-Saharan African epidemiological conditions. Their cost-sensitive framework prioritized sensitivity, reflecting real-world screening needs, and demonstrated that Extreme Gradient Boosting (XGB) achieved the highest predictive performance (AUC ≈ 0.956), marginally outperforming an enhanced Bayesian logistic regression model. While the study highlights the potential of ensemble models for malaria screening, the authors emphasized the need for real-world clinical validation beyond synthetic data.

In Nigeria-specific contexts, McLaughlin et al. [7] developed and validated supervised ML clinical assessment algorithms for malaria diagnosis in under-five children in Kano State. Using paired symptom data, patient location, and rapid diagnostic test (RDT) outcomes, their Random Forest–based model achieved improved sensitivity and specificity compared with standard Integrated Management of Childhood Illness (IMCI) protocols. This work demonstrates the practical utility of ML-enhanced clinical decision support systems in low-resource settings.

More recently, Awe et al. [8] explored ensemble ML models combined with explainable artificial intelligence (XAI) techniques for malaria diagnosis using clinical data from a Nigerian medical center. Among Random Forest, AdaBoost, Gradient Boosting, XGBoost, and CatBoost, Random Forest achieved the highest diagnostic performance (AUC ≈ 0.869). Importantly, the integration of explainability methods such as SHAP and LIME enabled identification of influential clinical features, enhancing model transparency and trustworthiness in clinical decision-making.

The integration of socio-economic factors with clinical outcomes has gained increasing attention. Ashaolu et al. [9] applied ML techniques to childhood malaria surveillance in Nigerian internally displaced person (IDP) camps. Using socio-demographic variables, household conditions, caregiver practices, and RDT results, their Random Forest model achieved the best predictive performance (AUC ≈ 0.892). The study identified modifiable socio-economic determinants such as caregiver education and occupation as key predictors of malaria positivity, highlighting the importance of social context in malaria risk modeling.

Similarly, Mthethwa and Melesse [10] analyzed the 2021 Nigeria Malaria Indicator Survey (NMIS) using multiple ML classifiers. Their findings showed that Random Forest and Support Vector Machine (SVM) models provided the most robust predictions of malaria infection among under-five children, outperforming Naïve Bayes, k-nearest neighbors, and decision tree models. This large-scale, nationally representative analysis underscores the value of combining clinical RDT outcomes with household-level socio-economic variables for population-level malaria prediction.

Ayoka and Nnadi [11] further extended this perspective by predicting malaria prevalence using Nigeria Demographic and Health Survey (DHS) data combined with geospatial covariates. Rather than binary classification, their study employed regression-based ML models, demonstrating that a Random Forest Regressor achieved near-perfect fit (R² ≈ 0.994). This approach illustrates the capability of ML models to estimate malaria burden at fine spatial and demographic resolutions, supporting proactive public health planning.

Weather and climatic factors remain critical drivers of malaria transmission. Pillay et al. [12] introduced a high-resolution, climate-informed malaria prediction framework using 23 years of daily incidence data and meteorological variables. By applying a deep learning Transformer model, the study demonstrated substantial improvements over XGBoost and traditional statistical models, achieving approximately 98% accuracy and an AUROC of around 80%. The results emphasize the ability of deep sequence models to capture complex temporal dependencies between climate variability and malaria incidence.

At a regional scale, Khan et al. [13] developed a district-level malaria outbreak prediction model for The Gambia by integrating historical meteorological data with non-climatic contextual factors. Among eight ML algorithms evaluated, XGBoost and C5.0 decision trees achieved the highest accuracy (≈93.3%), reinforcing the effectiveness of ensemble tree-based methods when climatic and contextual data are jointly considered.

In Nigeria, Oloyede [14] focused on malaria outbreak forecasting in Sokoto State using climatic variables such as rainfall and temperature. Using lagged time-series data, SVM outperformed Random Forest and k-nearest neighbors in predicting outbreak months up to three months in advance. Although the study did not incorporate socio-economic factors, it demonstrated the feasibility of climate-driven ML early warning systems in high-burden northern Nigerian regions.

Deep learning–based image analysis has emerged as a powerful tool for malaria diagnosis. Hemachandran et al. [15] evaluated convolutional neural network (CNN) architectures for classifying parasitized and healthy red blood cells using microscopic blood smear images. Among the tested models, MobileNetV2 achieved the highest accuracy (≈97.06%), outperforming deeper architectures such as ResNet50. Our study confirms the suitability of lightweight CNN models for automated malaria diagnosis, particularly in resource-constrained settings where computational efficiency is critical.

Previous malaria prediction studies have predominantly relied on climate-driven early warning systems or clinically intensive data sources, including meteorological variables, time-series incidence records, laboratory-confirmed diagnostics, and microscopic blood smear images. While such approaches are effective for seasonal forecasting or laboratory-supported diagnosis, they are less suited for immediate, point-of-care decision-making, particularly in low-resource settings where diagnostic delays, data unavailability, or rapid diagnostic test (RDT) failures are common. Climate-based models, although valuable for population-level risk monitoring, do not address the critical clinical gap created when symptomatic individuals receive false-negative RDT results and require timely triage before confirmatory testing becomes available.

In contrast, prior studies utilizing Demographic and Health Survey (DHS) or Malaria Indicator Survey (MIS) data have largely focused on descriptive prevalence estimation, regression-based association analysis, or geographically aggregated risk mapping. Only a limited number of DHS-based studies have explored machine learning–driven prediction, and these typically emphasize single-model performance, regional prevalence estimation, or the inclusion of geospatial covariates rather than actionable individual-level screening. Moreover, systematic evaluations of automated machine learning (AutoML) frameworks on nationally representative DHS/MIS data remain scarce, particularly with respect to model robustness, feature-driven risk stratification, and subgroup-specific behavior.

To contextualize these gaps and underscore the unique contributions of the present study, Table 1 contrasts key methodological aspects of recent malaria risk analyses (2023–2025) using Nigerian survey data, including NMIS 2021. While prior works effectively identify socio-demographic determinants or achieve moderate predictive accuracy (~79–80%) via traditional statistical or ML approaches, they predominantly apply uniform nationwide modeling without automated frameworks or explicit handling of spatial heterogeneity and bias limitations that hinder scalability, equity, and non-clinical early screening highlighted in the introduction. In contrast, this study pioneers H2O AutoML for robust, recall-oriented prediction against benchmarks, complemented by agglomerative hierarchical clustering (AHC) to mitigate bias and enable subgroup-specific tailoring, while identifying influential features from readily available household/demographic variables to support timely triage when RDTs fail or confirmatory testing is delayed.

**Table 1:** Comparison of Recent Studies on Malaria Risk Analysis and Prediction Using Survey Data in Nigeria.

Source	Dataset	Population	Feature Type	Methodology	AutoML	Bias/ Heterogeneity Handling	Prediction/ Outcome
[16]	NMIS 2021	Children <5	Residence, wealth index, mother’s education, gender	Statistical analysis (LR)	✗	✗ (nationwide pooled analysis)	Risk factors for severe complications
[17]	NMIS 2021	Children <5	Socio-demographic, household	Statistical analysis (Chi-square, LR)	✗	✗ (nationwide pooled analysis)	Determinants & associations
[18]	NMIS 2021	Children <5	Socio-demographic, household	RF, CatBoost, XGB with SMOTE for imbalance	✗	✗ (single nationwide model)	Individual-level prediction
[10]	NMIS 2021	Children <5	Socio-demographic, household	RF, SVM, DT, SLR, NB, KNN with SMOTE	✗	✗ (single nationwide model)	Individual-level prediction
[19]	DHS 2015–2020	General/ Children	Socio-economic + geospatial	Random Forest Regressor	✗	✗ (geospatial focus, regional effects)	Prevalence mapping
[9]	DHS/ IDP survey	Children	Socio-demographic	RF	✗	✗ (localized focus)	Individual prediction
[12]	National time-series	Population-level	Climate, incidence	DL (Transformer)	✗	✗ (aggregate forecasting)	Population forecasting
Our study	NMIS 2021	Children <5	Non-clinical	AutoML (H2O) + ML benchmarks (LR, DT, SVC, XGB)	✓	✓ (AHC-based clustering on non-regional features)	Individual & subgroup-level screening

This study addresses these gaps by introducing a nationally scalable, non-clinical, machine learning–based screening framework using the Nigeria Malaria Indicator Survey (MIS) 2021 dataset. Unlike climate-focused models or clinically dependent approaches, the proposed methodology leverages readily available household, demographic, and socio-economic variables to support early malaria risk identification when laboratory diagnostics are delayed or unreliable. Furthermore, this work represents one of the first comprehensive evaluations of AutoML (H2O AutoML) against rigorously tuned traditional machine learning models on DHS-MIS data, complemented by feature selection, spatial clustering, and cluster-specific modeling. By demonstrating how automated and interpretable ML frameworks can operate using routinely collected survey data, this study contributes a novel, deployment-oriented perspective to malaria screening and decision support in resource-constrained healthcare settings.

## Methodology

### Ethics statement

This study is a secondary analysis of de-identified, publicly available data from the nationally representative Nigeria Malaria Indicator Survey (MIS) 2021, with the sample restricted to children under 60 months of age. The MIS 2021 protocol was reviewed and approved by the National Health Research Ethics Committee of Nigeria (NHREC) and the ICF Institutional Review Board, and informed consent was obtained from a parent or guardian for each child’s participation and biomarker testing during the original survey. The authors received written authorization from The DHS Program to use the dataset (granted 20 January 2023) and analyzed it solely for statistical reporting in accordance with the DHS terms of use. Because the data were fully anonymized, contained no identifying information, and were already publicly available, this analysis did not constitute human subjects research and required no additional ethical approval. The study posed no risk to participant privacy.

### Proposed farmwork

**Fig 1:**
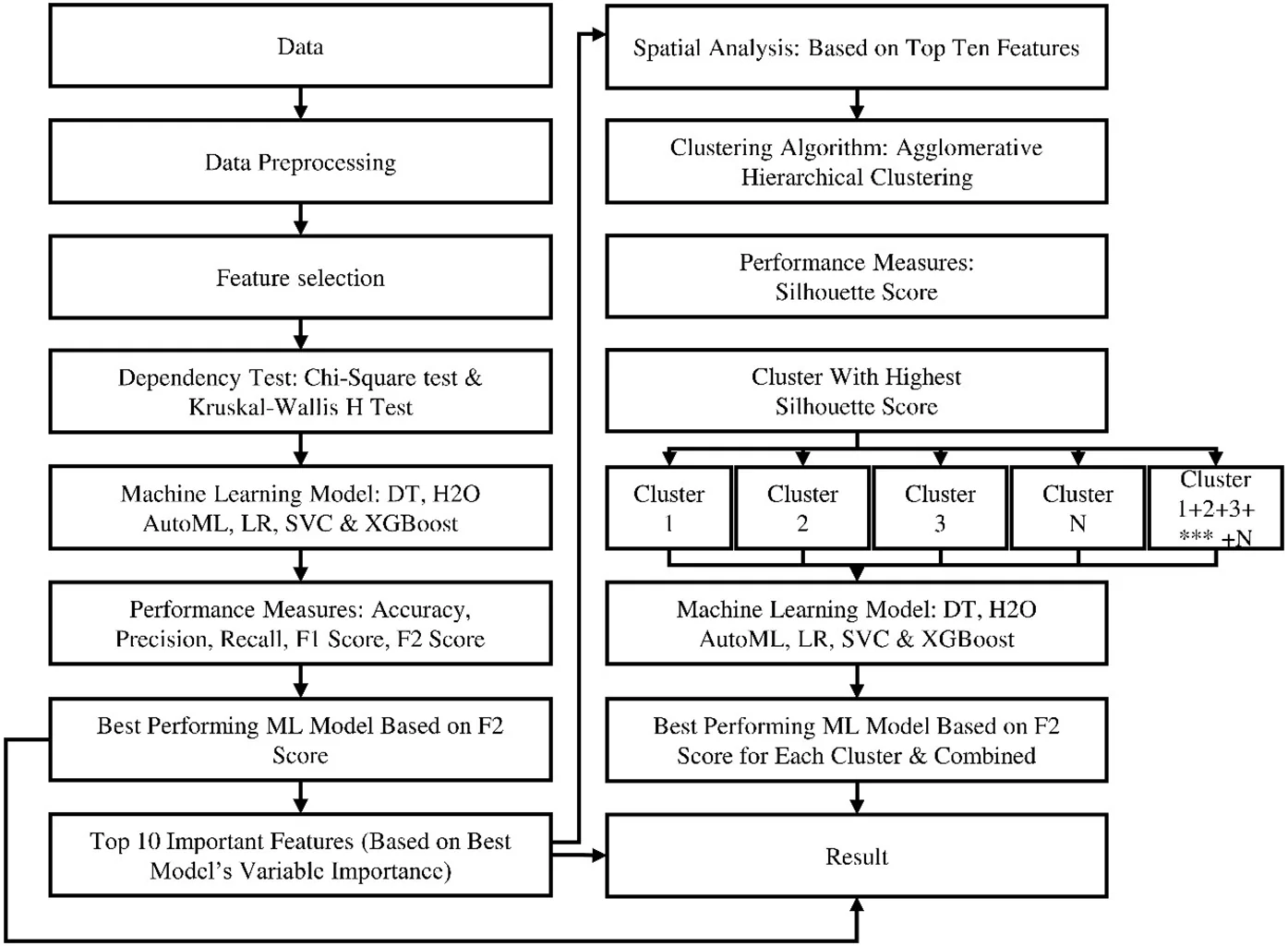
Working methodology of this study.

This study follows a structured and systematic methodological framework shown in Fig 1 consisting of two distinct analytical phases. In the first phase, machine learning and automated machine learning models are developed to predict malaria outcomes at the national level using the Nigeria Malaria Indicator Survey (MIS) 2021 dataset, following rigorous data preprocessing, feature selection, and statistical dependency analysis. The second phase focuses on spatial analysis, where clustering techniques are employed to segment the population into homogeneous subgroups, followed by cluster-specific malaria prediction

To capture regional and demographic heterogeneity. The complete methodological workflow, encompassing both the nationwide prediction and the spatially stratified modeling phases, is illustrated in Fig 1.

### Data source

The data used in this study is from a survey named Malaria Indicator Survey (MIS) of year 2021 of Nigeria [4]. Malaria Indicator Survey (MIS) is developed by The Monitoring and Evaluation Working Group (MERG) of Roll Back Malaria; they coordinate global efforts to combat malaria. They conduct a stand-alone household survey called the Malaria Indicator Survey (MIS) to collect data from a representative sample of respondents at the national and regional/provincial levels. The DHS Program plays a major role in the development and implementation of the MIS, including co-chairing the MERG Survey and Indicator Guidance Task Force, contributing to the development of the MIS package (questionnaires, manuals, and guidelines based on Demographic and Health Surveys materials), and maintaining a website that provides information and data for Malaria Indicator Surveys worldwide. They also provide standardized malaria indicators for nearly 30 countries [20]. The dataset used in this research was accessed on January 20, 2023, following approval of a formal data request submitted to the DHS Program. The publicly available dataset is fully anonymized; no personally identifiable information was accessible to the authors at any stage of data collection or analysis.

#### Survey design.

The Nigeria Malaria Indicator Survey (MIS) 2021 was conducted using a nationally representative multistage stratified cluster sampling design. The objective of this study was to develop and evaluate predictive machine learning models for individual-level malaria classification rather than to estimate nationally representative epidemiological parameters. Accordingly, the machine learning models were developed using individual-level observations without incorporating survey sampling weights, survey strata, or primary sampling unit (PSU) information into model training. Model evaluation was performed by partitioning the dataset into training and testing sets using stratified train–test splitting based on the malaria outcome variable, followed by nested cross-validation to ensure robust and unbiased performance assessment.

### Data description

The dataset consists of 70428 observations and 225 features. Initially, 43 features were selected for predictive analysis, including variables with missing (***NaN***) values. These features primarily represented socioeconomic, household, and illness-related characteristics that could be identified through observable physical signs. After that, the dataset was filtered with the “children age” below 60 months to drive the study to **minors** of the Nigeria demographic. Descriptions of the encoded data of selected features are taken from *Demographic and Health Surveys Standard Recode Manual for DHS7* [21].

**Fig 2:**
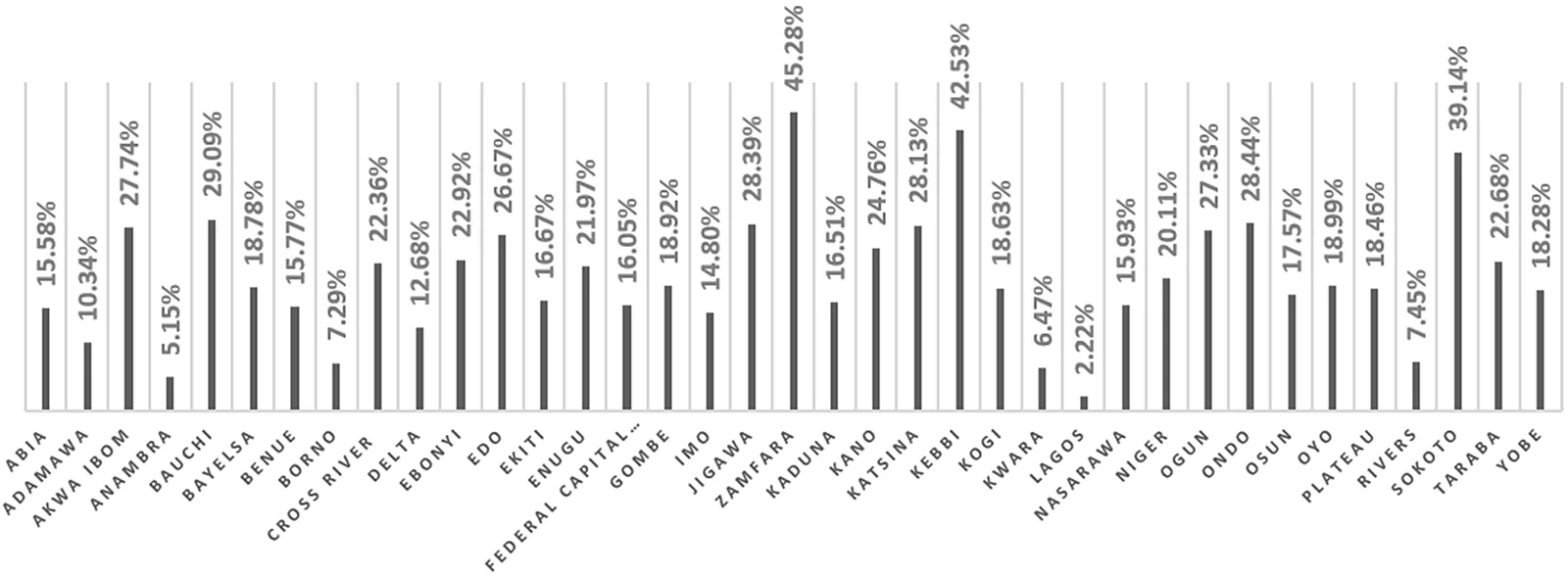
State wise Malaria Positive Serum test results of minors.

Here in Fig 2 state wise Malaria Positive percentage based on serum test is shown where Lagos has minimum positive cases and Zamfara has maximum positive cases.

### Data preprocessing

After filtering the data for children under five and removing observations with missing target values, features with insufficient data were excluded to ensure data completeness and model stability. The remaining predictors were then tested for their association with the target variable, “Final result of malaria from blood smear test,” using the Chi-square test for categorical variables and the Kruskal–Wallis’ test for continuous variables. Based on the p-values reported in Table 2, two features with p-values > 0.05 were excluded, and the remaining significant predictors were retained for model development. Consequently, the final predictive model was developed using 13 predictor variables and 10,655 observations, comprising 78.75% malaria-negative and 21.25% malaria-positive cases. The dataset was partitioned into training (80%) and testing (20%) sets using stratified train–test splitting based on the malaria outcome variable to preserve class distribution. To address class imbalance while preventing information leakage, the SMOTENC oversampling technique was applied only to the training dataset. The independent testing dataset remained unchanged and was used exclusively for model evaluation.

**Table 2:** Correlation between predictor features and target feature.

SL	Features	P-Value	Data type	Decision
1	Number of household members	0.00E + 00	int64	YES
2	SubRegion	1.54E-105	category	YES
3	Type of place of residence	1.15E-55	category	YES
4	Native language of respondent	7.41E-41	category	YES
5	Source of drinking water	1.69E-59	category	YES
6	Time to get to water source (minutes)	0.00E + 00	int64	YES
7	Type of toilet facility	3.09E-86	category	YES
8	Has mosquito bed net for sleeping	7.58E-01	category	NO
9	Owns land usable for agriculture	4.63E-49	category	YES
10	Owns livestock, herds or farm animals	3.55E-32	category	YES
11	Owns pigs	2.67E-257	int64	YES
12	Wealth index combined	1.10E-116	category	YES
13	Region	9.51E-52	category	YES
14	Child’s age in days	0.00E + 00	int64	YES
15	Sex of the Child	8.76E-01	category	NO
16	Final result of malaria from blood smear test	0.00E + 00	category	YES

### Machine learning algorithms

For predictive analysis four traditional Machine Learning (ML) and one Automated Machine Learning framework H2O are implemented. The algorithms are as follow

#### Decision tree classifier.

Decision trees are a popular and effective tool used in various fields, including medicine and pathology [22]. They work by iteratively dividing the data into subspaces based on attribute values, assigning the most appropriate class to the leaves that represent those values [23]. Unlike other supervised learning models, decision trees can be used for both classification and regression problems. Their goal is to create a model from past data that generates simple decision rules for predicting the class or value of the target variable [24]. To classify data using decision trees, the process involves several steps:

i Calculating the Gini index,
ii Evaluating and selecting the best split by splitting the dataset, and
iii Generating the decision tree by considering terminal nodes, recursive nodes, and developing the tree.

#### H2O AutoML.

Automated machine learning (AutoML) has been used by researchers to address the challenge of model optimization by automating the end-to-end machine learning pipeline, including data preparation, feature engineering, model generation, hyperparameter optimization, and performance evaluation [25]. A common strategy employed by AutoML frameworks is Bayesian optimization, which combines meta-learning with sequential model-based optimization to iteratively improve model performance [26]. Several widely used Python-based AutoML frameworks, including Auto-Sklearn, TPOT, Auto-Keras, H2O.ai, and Google’s AutoML, support automated machine learning model development [27].

In this study, the H2O AutoML framework was used. H2O is a distributed machine learning platform designed to efficiently process large datasets and includes an AutoML framework that automatically trains, tunes, and ranks multiple machine learning models without requiring manual candidate selection. AutoML generates a leaderboard of candidate models based on predefined performance metrics, enabling systematic comparison of model performance. H2O AutoML further improves predictive performance by combining automated hyperparameter optimization with ensemble learning techniques [28,29].

The dataset was initially partitioned into training (80%) and testing (20%) sets using stratified train–test splitting based on the malaria outcome variable. To address class imbalance while preventing information leakage, the SMOTENC oversampling technique was applied only to the training dataset, whereas the independent testing dataset remained unchanged and was used exclusively for model evaluation. H2O AutoML was then configured to generate a maximum of 10 candidate models using 10-fold internal cross-validation with a fixed random seed (123). Model ranking and early stopping were based on the Area Under the Precision–Recall Curve (AUCPR), with three stopping rounds and a stopping tolerance of 0.001. To ensure a fair and consistent comparison across all machine learning algorithms, the same SMOTENC-balanced training dataset was used for H2O AutoML and the conventional machine learning models. Consequently, H2O AutoML’s optional internal class-balancing mechanism (balance_classes) was not enabled because class imbalance had already been addressed during preprocessing of the training data. Deep Learning and Stacked Ensemble algorithms were excluded from the AutoML search space to facilitate direct comparison with the individual machine learning algorithms evaluated in this study while reducing computational complexity. All remaining parameters were retained at their default H2O AutoML settings. Following model training, H2O AutoML generated a comprehensive leaderboard ranking candidate models according to their predictive performance, from which the highest-performing models were selected for subsequent evaluation and comparison.

#### Logistic regression.

Logistic Regression (LR) is a supervised machine learning algorithm widely used for binary classification and disease prediction because of its simplicity, interpretability, and computational efficiency [30]. The algorithm estimates the probability of a binary outcome by modeling the relationship between the predictor variables and the target variable using the logistic function [31–34].

In this study, LR was employed as a baseline classifier to predict malaria status. The mathematical representation of LR is shown in Equation (1), where *q(d = 1)* denotes the probability of the positive class, $a_{\text{1}}$*,* $a_{\text{2}}$*,...,* $a_{k}$ represent the predictor variables, and $\alpha_{\text{1}}$*,* $\alpha_{\text{2}}$*,...,* $\alpha_{k}$ are the estimated regression coefficients.


$$\begin{array}{c} og\frac{q(d=\text{1})}{\text{1}-q(d=\text{1})}=\alpha_{\text{0}}+\alpha_{\text{1}}a_{\text{1}}+\alpha_{\text{2}}a_{\text{2}}+\cdots +\alpha_{k}a_{k},\;where\;k\;=\;\text{1},\;\text{2},\;...,\;n \end{array}$$ (1)

#### Support vector classifier.

Support Vector Classifier (SVC) is a supervised machine learning algorithm widely used for disease prediction because of its ability to effectively classify high-dimensional data while minimizing generalization error [35,36]. SVC constructs an optimal separating hyperplane that maximizes the margin between classes, thereby improving classification performance and robustness.

The mathematical formulation of the SVC classifier is presented in Equations (2) to (4). For a training sample (*α*_*i*_*, β*_*i*_), where the class label *β*_*i*_ ∈ {−1, + 1}, the classification constraints are defined as:


$$\begin{array}{c} If\;\beta_{i}=\;+\text{1};\;\gamma \alpha_{i}\;+\;\theta \;\geq \text{1} \end{array}$$ (2)

$$\begin{array}{c} If\;\beta_{i}=\;-\text{1};\;\gamma \alpha_{i}+\;\theta \;\leq -\text{1} \end{array}$$ (3)

The constraints can be combined into the standard SVC formulation:


$$\begin{array}{c} \beta_{i}(\;\gamma^{T}\alpha_{i}+\;\theta )\;\geq \;\text{1} \end{array}$$ (4)

where *γ* represents the weight vector, *θ* is the bias term, *α*_*i*_ denotes the predictor variables, and *β*_*i*_ is the corresponding class label. The optimal hyperplane is determined by maximizing the margin between the two classes while satisfying these constraints.

#### EXtreme gradient boosting algorithm.

XGBoost is a modern machine learning algorithm introduced in February 2014. It has gained widespread attention due to its strong predictive performance and efficient training speed. XGBoost is an enhanced version of the Gradient Boosting Decision Tree (GBDT) algorithm and can be applied to both classification and regression tasks. Notably, XGBoost belongs to the family of boosting tree algorithms, which combine multiple weak learners to form a strong classifier. The tree model used in XGBoost is the Classification and Regression Tree (CART).

The core idea of the algorithm is to iteratively add decision trees, where each new tree is trained to fit the residual errors of the previous prediction. After training **k** trees, the final prediction for a sample is obtained by summing the output scores from all trees based on the sample’s features. The workflow of the algorithm can be summarized as follows:

i The first step is to calculate the first and second derivative matrices of the loss function for each sample before adding new trees.
ii In each iteration, a new tree is added to fit the residual from the previous tree.
iii The optimal split point is determined by calculating the split gain value of the objective function and using a greedy algorithm to find the best tree structure.
iv To prevent overfitting, a new tree is added to the model and scaled by a factor. A step size or learning rate is used when fitting residuals to control optimization and provide more room for future learning.
v After training, a model consisting of multiple trees is obtained, where each tree has several leaf nodes.
vi Samples fall on different leaf nodes in each tree based on their feature values. The final prediction is calculated by multiplying the score of each tree’s corresponding leaf node by the tree’s weight.

In terms of model representation, if we iterate for *t* rounds, meaning we generate *t* residual trees, the model’s predicted value can be expressed as follows (Equation (5)):


$$\begin{array}{c} {\hat{y}}_{i}=\sum_{t=\text{1}}^{T}f_{t}(x_{i}),f_{t}\in F \end{array}$$ (5)

In this expression, $f_{t}(x_{i})$ represents the predicted value of the *t*^*th*^ residual tree for the *t*^*th*^ residual of $x_{i}$. ${\hat{y}}_{i}$ represents the model’s predic*t*ed value, $f_{t}$ is the number of residuals in the *t*^*th*^ round, and *F* is the function space of the residual tree. The loss function is also an essential component of this algorithm, with error sources mainly including training errors and model complexity. The primary formula for the loss function is as follows (Equation (6)):


$$\begin{array}{c} obj=\sum_{i=\text{1}}^{n}l(y_{i},{\hat{y}}_{i})+\sum_{k=\text{1}}^{K}\Omega (f_{k}) \end{array}$$ (6)

In this formula, l represents the loss function and Ω is the regularization term. For the *t*^*th*^ round of training, the loss expression above satisfies the following relationship (Equation (7) & (8)):


$$\begin{array}{c} {\hat{y}}_{i}^{t}={\hat{y}}_{i}^{t-\text{1}}+f_{i}(x_{i}) \end{array}$$ (7)

$$\begin{array}{c} \Omega (f_{t})=(\sum_{k=\text{1}}^{T-\text{1}}\Omega (f_{k}))+\Omega (f_{t})=const+\Omega (f_{t}) \end{array}$$ (8)

As a result, the loss for the *t*^*th*^ round, combined with the second-order Taylor expansion, can be simplified into the following form (Equation (9)):


$$\begin{array}{c} {obj}^{t}\approx \sum_{i=\text{1}}^{n}\left[l(y_{i},{\hat{y}}_{i}^{t-\text{1}})+g_{i}f_{i}(x_{i})+\frac{\text{1}}{\text{2}}h_{i}(f_{t}(x_{i}){)}^{\text{2}}\right]+\Omega \left(f_{t}\right)+const \end{array}$$

$$\begin{array}{c} =\sum_{i=\text{1}}^{n}\left[g_{i}f_{t}(x_{i})+\frac{\text{1}}{\text{2}}h_{i}(f_{t}(x_{i}){)}^{\text{2}}\right]+\Omega (f_{t})+const \end{array}$$ (9)

In the objective function, the parameters $g_{i}$ and *h*_*i*_ correspond to the *t*^*th*^ residual tree. By determining *t*hese parameters based on the number of leaf nodes, the function’s minimum value can be obtained. It’s important to note that the tree’s structure also affects the model’s optimal result. XGBoost uses a greedy algorithm to generate the tree’s specific architecture, starting at the root node and traversing all features. For continuous features, they are sorted in ascending order. This concludes our discussion of the XGBoost algorithm and how it works [37].

#### Bayesian optimization.

Bayesian Optimization (BO) was employed in this study to fine-tune the hyperparameters of four traditional machine learning models Logistic Regression (LR), Support Vector Machine (SVM), Decision Tree (DT), and XGBoost (XGB). BO is particularly effective for optimizing black-box functions that are expensive to evaluate, such as validation performance under repeated K-fold cross-validation. In high-dimensional hyperparameter spaces with complex conditional and categorical structures, Gaussian Process (GP) surrogates which are commonly used in classical BO often struggle with scalability and modeling flexibility.

To overcome these limitations, we adopted the **Tree-structured Parzen Estimator (TPE)** as the surrogate model, originally proposed by Bergstra et al. [38]. Unlike GP-based BO, which models the conditional distribution *p(y | x)*, TPE models the inverse density *p(x | y)* using non-parametric kernel density estimators (KDEs). This reformulation allows TPE to scale efficiently and naturally incorporate categorical and conditional hyperparameters.

#### TPE formulation.

Let (x) denote a hyperparameter configuration and y = f(x) be its associated objective value (e.g., validation loss or the negative F2-score). A threshold y* is chosen as a γ-quantile of the observed objective values such that Equation (10)


$$\begin{array}{c} p(y\;<\;y^{*})\;=\;\gamma \end{array}$$ (10)

TPE partitions the observations into two regions:

- **Good region** (top γ fraction of configurations) can be determined by Equation (11):

$$\begin{array}{c} \left(x)\;=\;p(x\;|\;y\;<\;y^{*}\right) \end{array}$$ (11)

- **Bad region** (remaining configurations) with Equation (12):

$$\begin{array}{c} g(x)\;=\;p(x\;|\;y\;\geq \;y^{*}) \end{array}$$ (12)

Both densities are modeled using kernel density estimation, enabling TPE to flexibly approximate complex distributions over hyperparameters.

#### Acquisition function.

Using Bayes’ rule and the Expected Improvement (EI) objective, Bergstra et al. [38] showed that the EI for TPE can be expressed as Equation (13),


$$\begin{array}{c} EI(x)\propto {\left(\;\gamma +(\text{1}-\gamma )\frac{g(x)}{l(x)}\right)}^{-\text{1}} \end{array}$$ (13)

Thus, maximizing EI is equivalent to selecting hyperparameters where the density under the good region l(x) is large and the density under the bad region g(x) is small, i.e., maximizing


$$\begin{array}{c} \frac{l(x)}{g(x)} \end{array}$$

This strategy naturally guides the search toward promising hyperparameter regions while avoiding exhaustive search.

Hyperparameter optimization was conducted using Optuna (Bayesian optimization via the TPE sampler) strictly within the training folds during cross-validation to prevent data leakage. The final selected hyperparameter configurations for all machine learning models (across the 14-feature dataset, Cluster 1, and Cluster 2) are provided in S1 Table.

#### Cross-validation protocol clarification.

To ensure a fair and robust evaluation of all predictive models, a consistent cross-validation protocol was applied throughout the study. To resolve the class imbalance issue mentioned in **Data Preprocessing** section (78.75% malaria-negative and 21.26% malaria-positive cases) *Synthetic Minority Over-sampling Technique for Nominal and Continuous features (SMOTENC)* technique was applied. Nested cross-validation were employed, in which Bayesian Optimization was conducted within the inner 10-fold cross-validation loop for hyperparameter tuning, while an independent held-out test set was reserved exclusively for final performance evaluation. This design prevents information leakage from the test data into the model selection process and provides an unbiased estimate of generalization performance. For the H2O AutoML framework, internal K-fold cross-validation was enabled using *nfolds = 10*. H2O AutoML performs automated model selection, hyperparameter optimization, and ensemble construction using only the training data and its internal cross-validation folds. The final leaderboard models were then evaluated on the same held-out test set used for traditional models, ensuring direct comparability across all approaches. This unified validation strategy guarantees methodological rigor and eliminates discrepancies arising from differing evaluation procedures.

## Technical setup and results

### Implementation of ML and H2O AutoML framework

To develop predictive models for malaria detection, this study utilized the most significant features identified in Table 2. These features were used to train both conventional machine learning (ML) models and the H2O AutoML framework with 80% train and 20% test split. Traditional machine learning models in this study were tuned using Bayesian Optimization under cross-validation, whereas the H2O AutoML framework performs hyperparameter optimization and model selection internally using its automated pipeline. Both approaches involve hyperparameter tuning; however, AutoML centralizes these steps within a unified framework rather than requiring explicit user intervention. In this study, the traditional ML models Logistic Regression (LR), Extreme Gradient Boosting (XGB), Decision Tree (DT), and Support Vector Classifier (SVC) were fine-tuned using Bayesian Optimization with *10-fold cross-validation* to ensure optimal performance. In contrast, the H2O AutoML framework was configured with predefined settings, allowing model selection and hyperparameter optimization to be handled internally without manual parameter specification. Once trained, the models were evaluated using multiple performance metrics, including accuracy, precision, recall, F1-score, and F2-score. Table 3 presents a comparison of these metrics. H2O GLM achieved the highest F_2_-score (79.38%) and recall (83.08%), indicating its superior ability to identify malaria-positive cases and its suitability for recall-oriented screening. It also demonstrated a minimal train–test accuracy gap of 0.59 percentage points (72.43% vs. 71.84%), suggesting consistent generalization between the training and test datasets. XGB achieved the second-highest F_2_-score (78.34%) and a comparable accuracy gap of 0.59 percentage points, while DT achieved the smallest positive accuracy gap (0.31 percentage points) but a lower F_2_-score (76.49%). LR showed a slightly higher test accuracy than training accuracy, but its F_2_-score was comparatively lower (71.67%).

**Table 3:** Performance Metrics of ML Models Trained on 13 Features.

Model	Train Accuracy	Test Accuracy	Precision	Recall	F1-score	F2-score
H2O GLM	72.43%	71.84%	67.38%	83.08%	74.41%	**79.38%**
LR	68.12%	68.50%	67.00%	72.94%	69.84%	71.67%
XGB	76.13%	75.54%	73.61%	79.62%	76.50%	78.34%
DT	70.36%	70.05%	66.92%	79.32%	72.59%	76.49%
SVC	75.16%	73.27%	71.14%	78.31%	74.55%	76.76%

**Fig 3:**
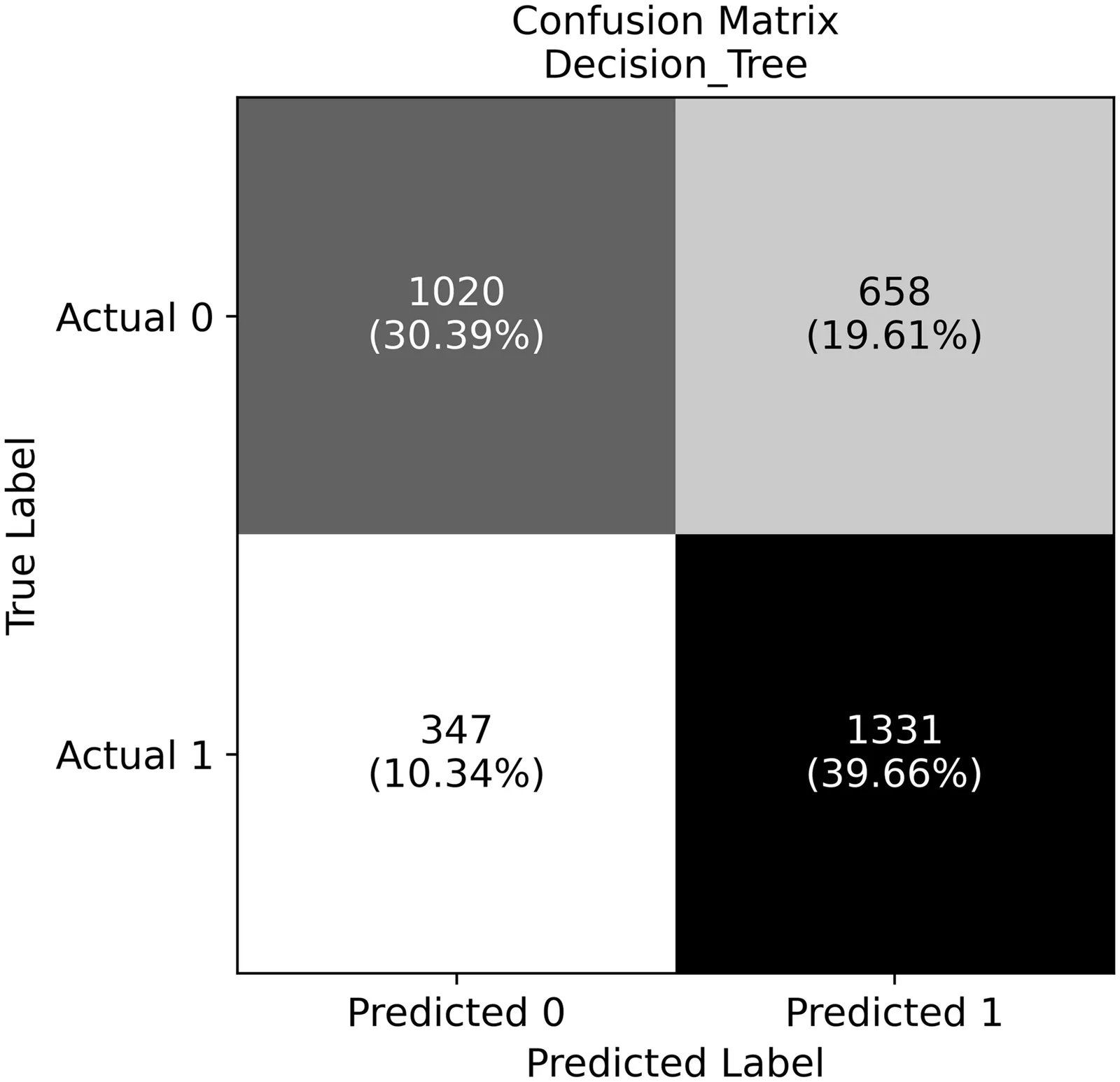
Confusion Matrix of DT.

**Fig 4:**
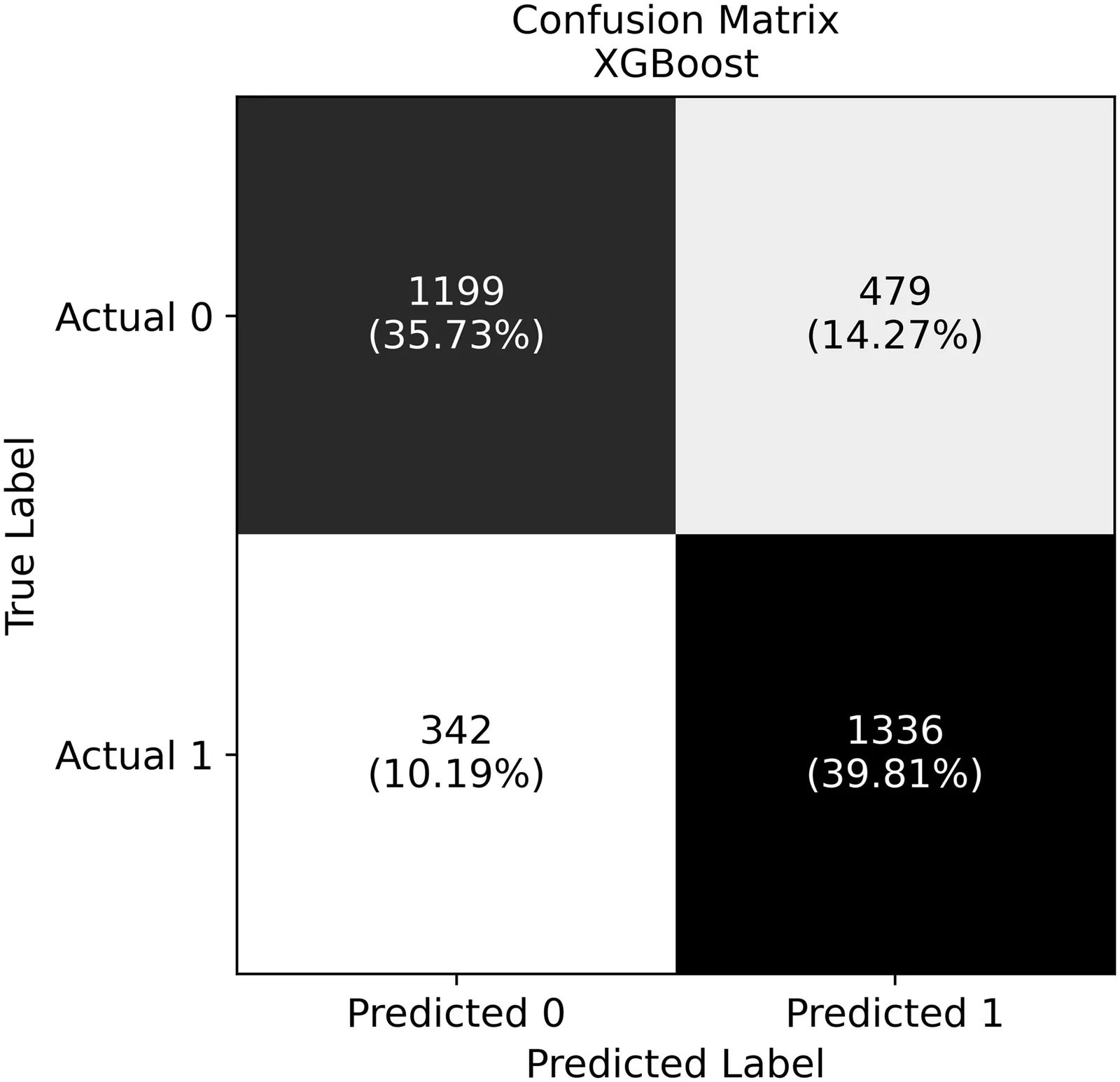
Confusion Matrix of XGB.

**Fig 5:**
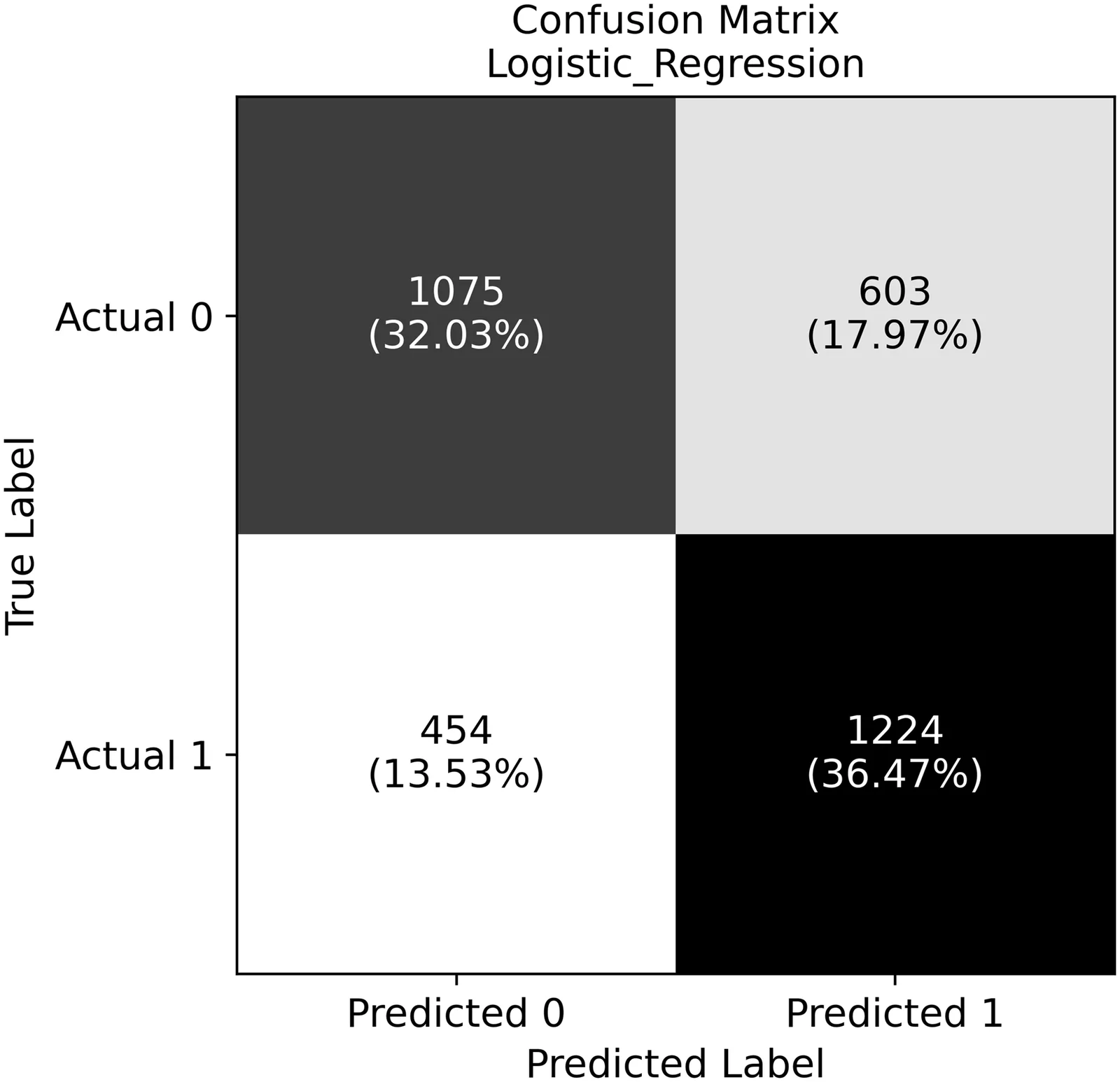
Confusion Matrix of LR.

**Fig 6:**
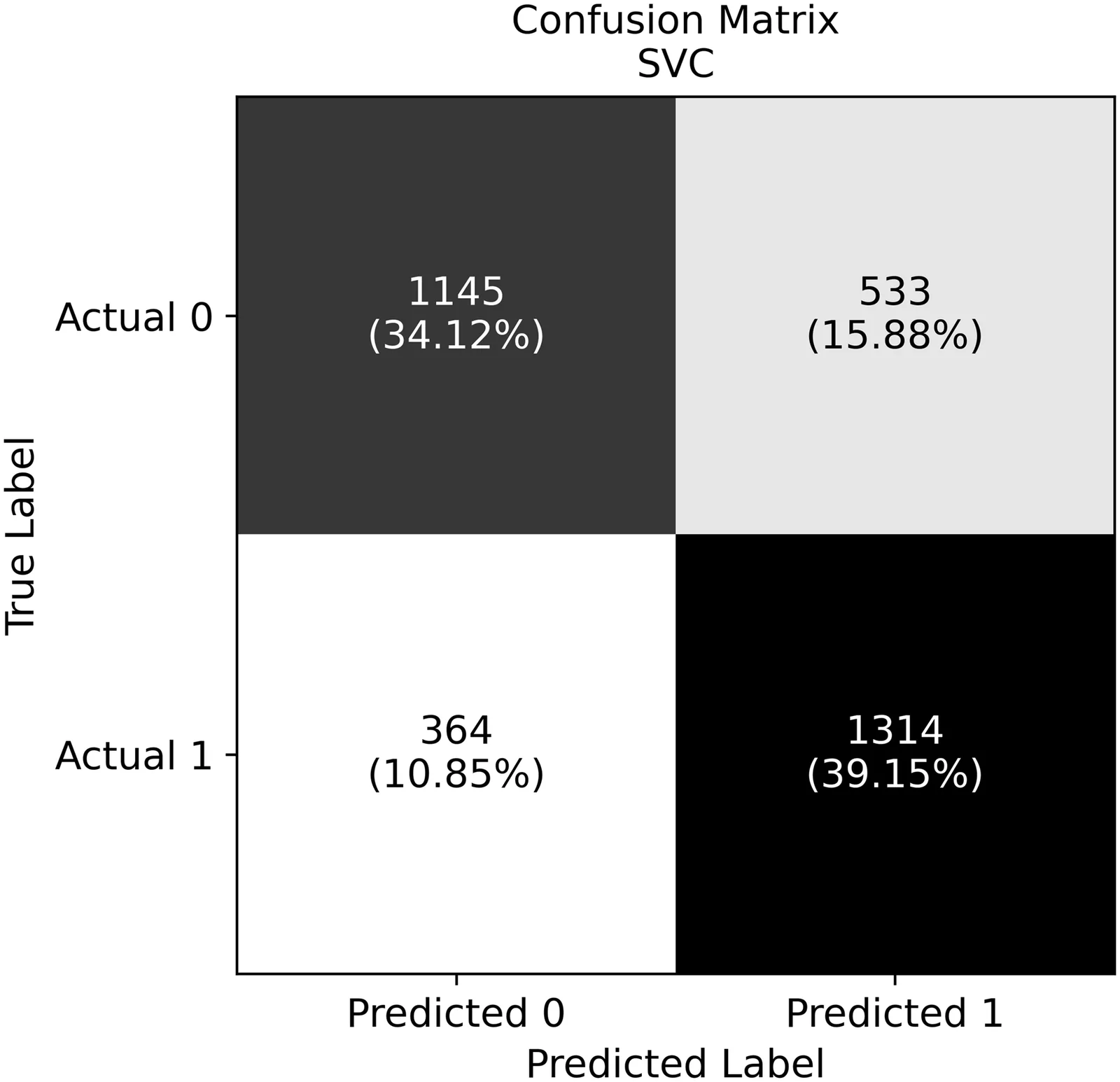
Confusion Matrix of SVC.

**Fig 7:**
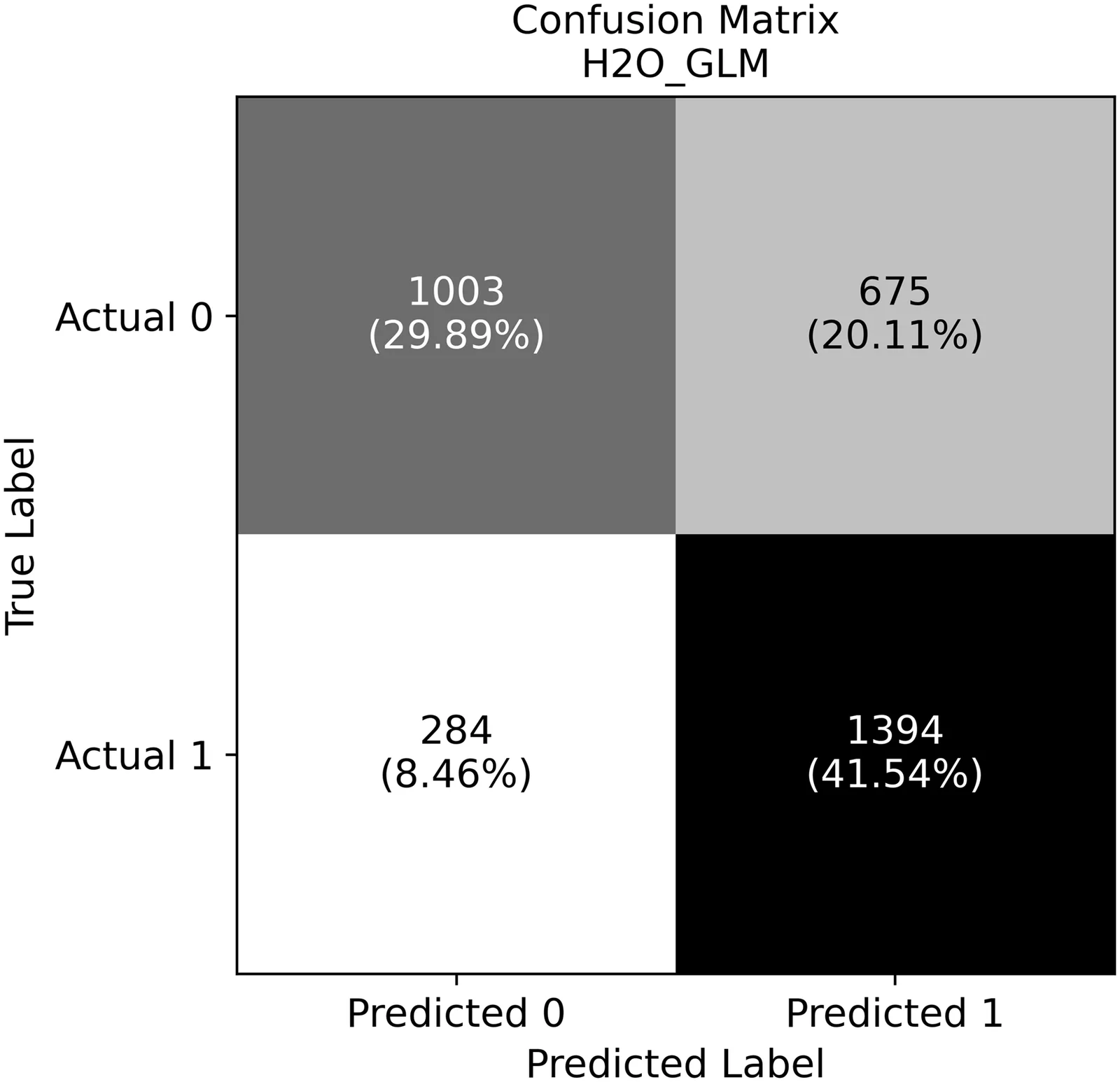
Confusion Matrix of H2O GLM.

**Fig 8:**
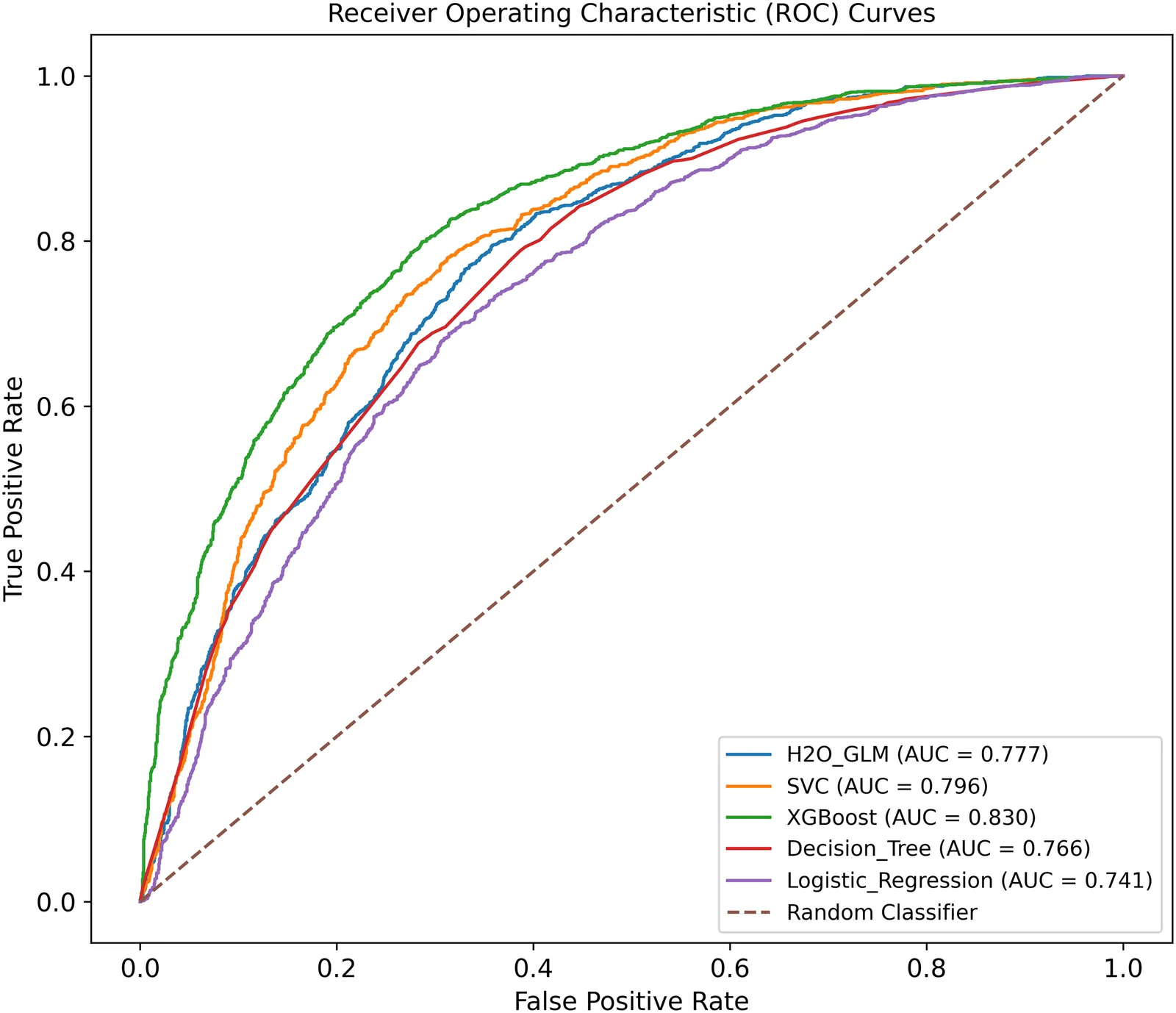
AUC-ROC Curve.

To further evaluate the classification performance and misclassification patterns of the models, confusion matrices were generated for all classifiers (Figs 3–7). The results show that H2O GLM achieved the highest number of correctly identified malaria-positive cases (true positives; 1,394) and the lowest number of false negatives (284), corresponding to the highest recall among the evaluated models. XGB correctly classified 1,336 malaria-positive cases with 342 false negatives, while DT identified 1,331 positive cases with 347 false negatives. SVC and LR recorded 1,314 and 1,224 true positives, with 364 and 454 false negatives, respectively. Regarding overall discriminative ability, the AUC-ROC analysis (Fig 8) showed that XGB achieved the highest AUC-ROC (0.830), followed by SVC (0.796), H2O GLM (0.777), DT (0.766), and LR (0.741). Although XGB demonstrated the strongest overall class-discrimination capability, H2O GLM provided the best recall-oriented performance, achieving the highest recall and F_2_-score while maintaining a minimal train–test accuracy gap. Therefore, given the primary objective of minimizing false-negative predictions in malaria screening, H2O GLM was selected as the most suitable model for this analysis.

**Fig 9:**
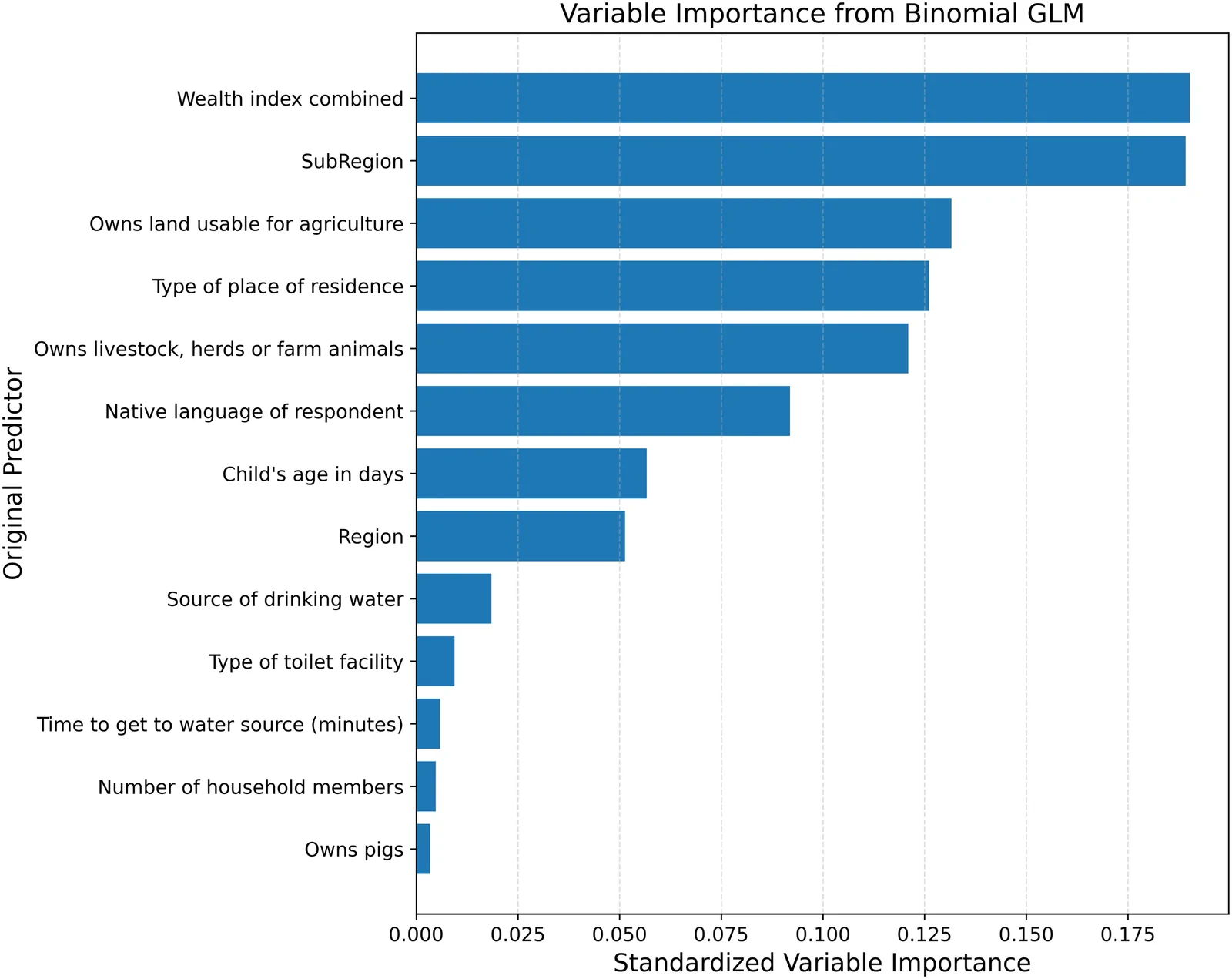
Variable Importance of GLM.

Lastly, Fig 9 illustrates the variable importance (VI) plot generated from the H2O GLM. Variable importance was quantified using the method proposed by Xie and Luo [39]. This method combines coefficient estimates and their standard errors to derive an importance score for each predictor, while aggregating the contributions of multiple dummy-coded levels for categorical variables using link-function-specific formulations. The VI plot indicates that the *Wealth index combined* and *SubRegion* were the most influential predictors of the target variable compared with the other features.

### Spatial analysis

Despite malaria being a major public health concern in Nigeria, there remains a significant gap in nationwide spatial analysis studies that explore the geographic and demographic patterns of malaria cases. While recent studies have demonstrated the value of spatial targeting for intervention delivery optimization [40], comparable spatial stratification approaches for diagnostic risk screening remain underexplored**.** To address this gap, Agglomerative Hierarchical Clustering (AHC) was employed in conjunction with Multiple Correspondence Analysis (MCA), a dimensionality reduction technique used to identify latent patterns and behavioral trends in malaria distribution across Nigeria.

Because the selected predictor variables comprised both categorical and continuous data types, pairwise dissimilarities were computed using Gower distance, which is suitable for mixed-type data. Agglomerative Hierarchical Clustering (AHC) was subsequently performed using the average linkage criterion based on the Gower distance matrix. To determine the optimal number of clusters, clustering solutions ranging from 2 to 10 clusters were evaluated, and the corresponding silhouette coefficients were calculated using the precomputed Gower distance matrix. The clustering solution with the highest silhouette coefficient was selected for subsequent subgroup analysis.

**Fig 10:**
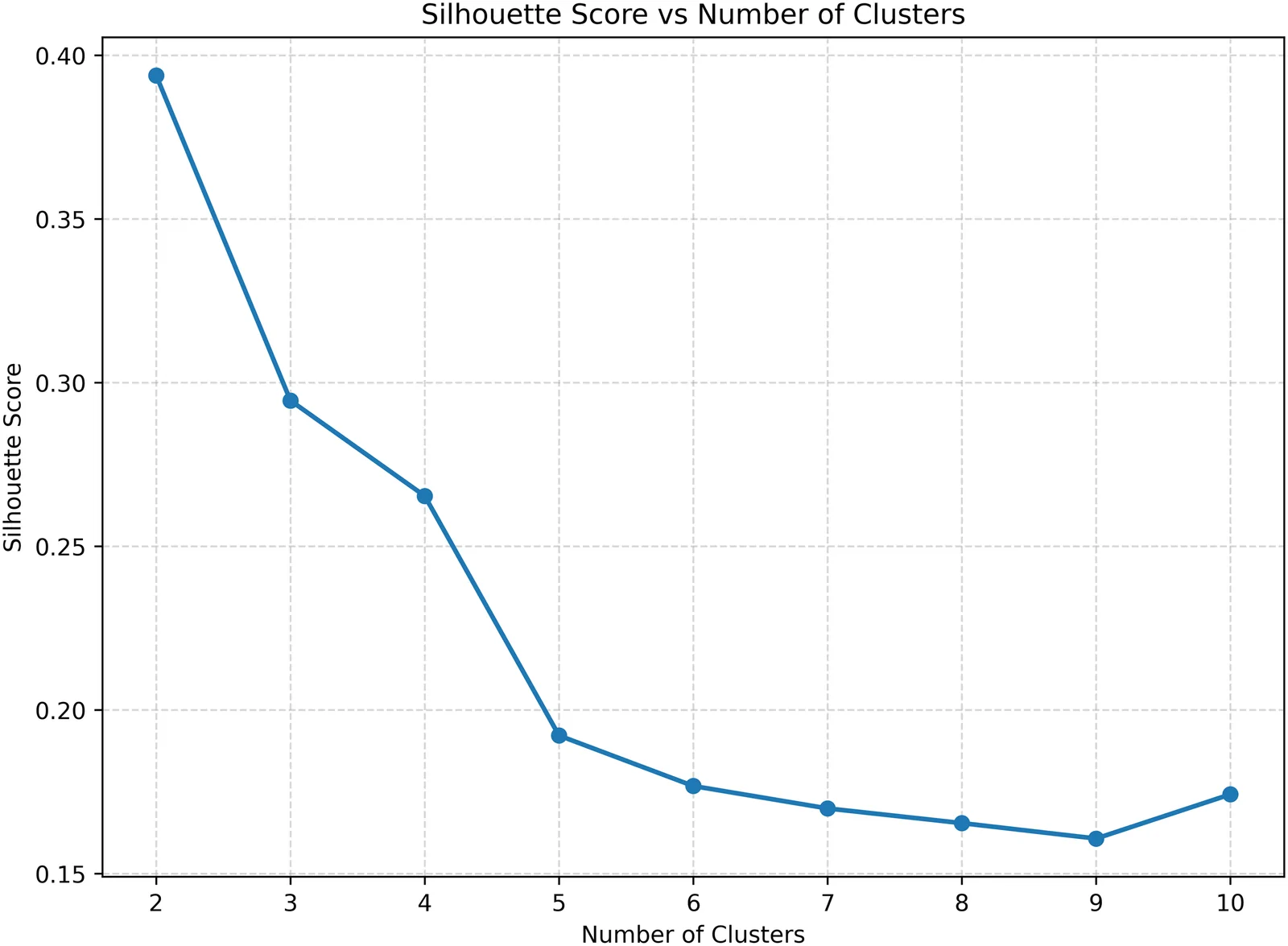
Silhouette Score vs. Number of Clusters in AHC.

**Fig 11:**
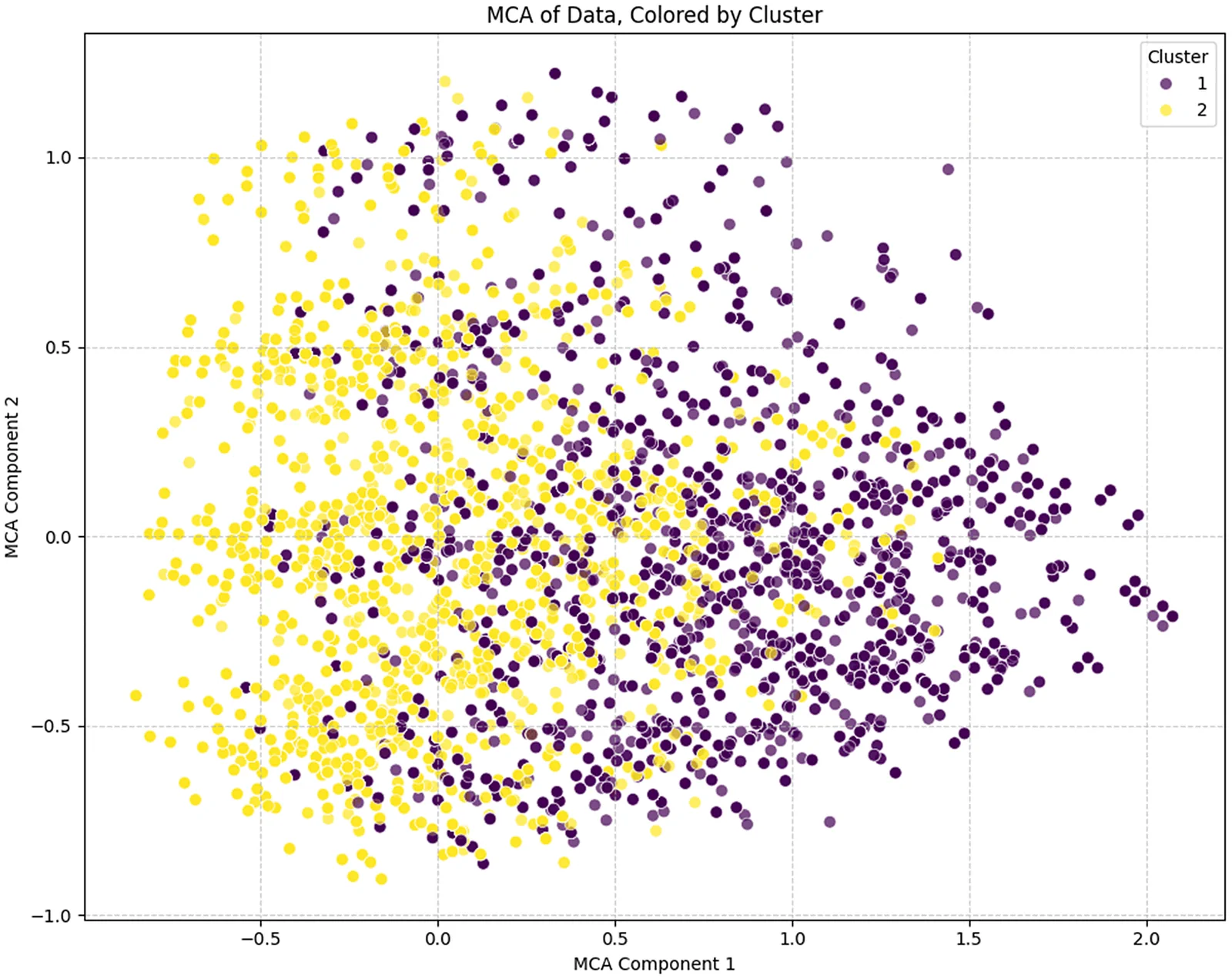
Two-dimensional MCA projection of observations colored according to the two clusters identified by agglomerative hierarchical clustering.

To minimize regional bias and place greater emphasis on other influential factors, the clustering algorithm was applied using the top eight key features from the variable importance (VI) plot, excluding “SubRegion” and “Region”. The AHC model was evaluated across a range of cluster numbers, from 2 to 10, to identify the optimal grouping based on clustering quality. The Silhouette Score was used as the evaluation metric. As illustrated in Fig 10, the highest Silhouette Score (0.3939) was obtained with a two-cluster solution. Fig 11 presents the corresponding Multiple Correspondence Analysis (MCA) projection, which shows partial overlap between the two clusters while also indicating differences in their overall distribution. Although the Silhouette Score indicates only modest cluster separation, the two-cluster solution represented the best-performing configuration among those evaluated and was therefore selected for subsequent subgroup analyses.

The relationship between the clustering results and the **Wealth Index Combined** the top most variable in VI plot further supports the selection of the two-cluster solution based on the highest silhouette score and is consistent with the variable-importance analysis.

**Fig 12:**
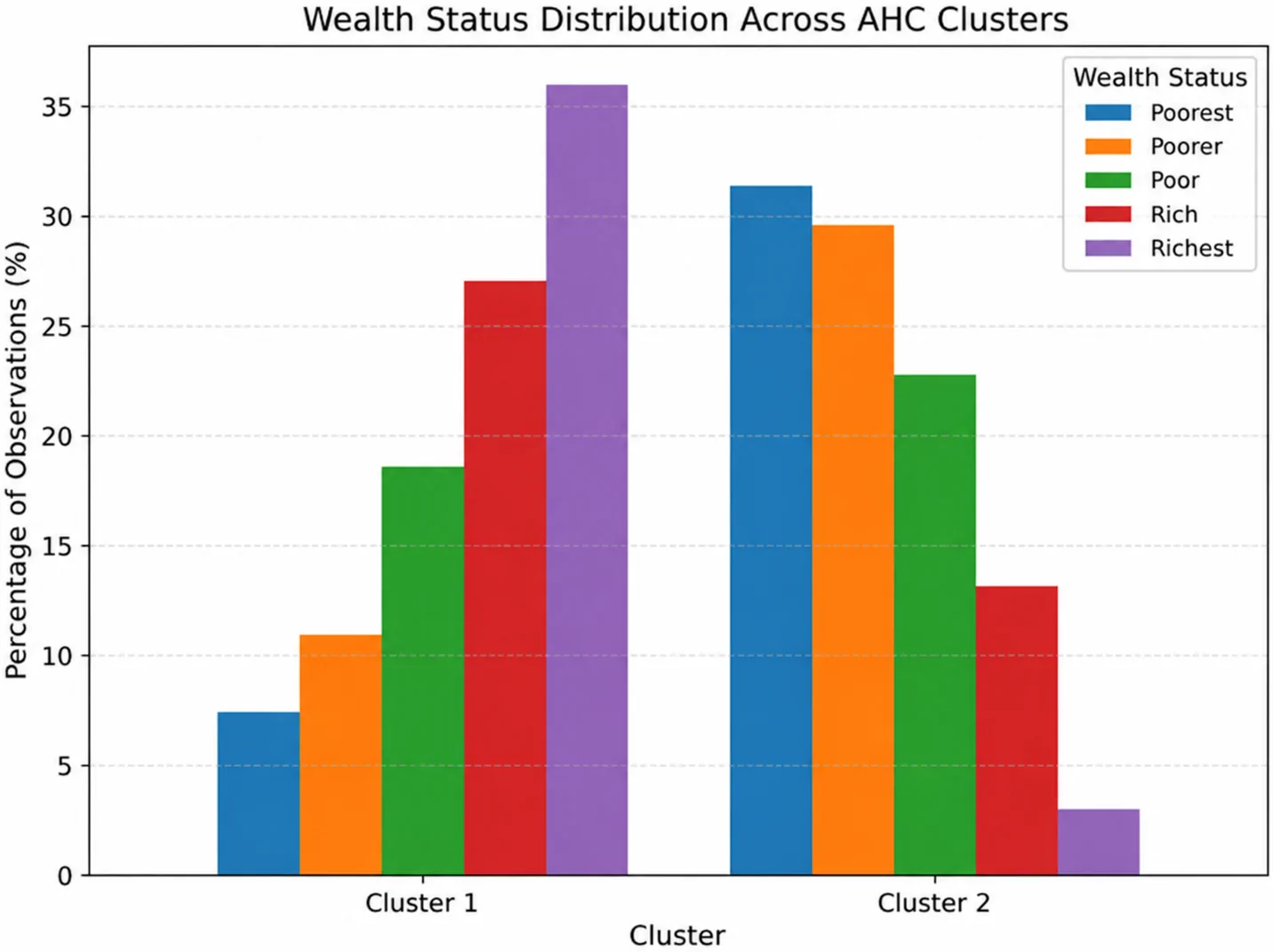
Wealth Index Combined Distribution Across AHC Clusters.

As shown in Fig 12, *Cluster 1* contains a higher proportion of individuals classified in the Richest and Richer wealth categories, with relatively fewer individuals in the Poorest and Poorer categories. In contrast, *Cluster 2* comprises a higher proportion of individuals in the Poorest and Poorer categories and fewer in the Richest and Richer categories. The proportion of individuals in the Poor wealth category is relatively similar across both clusters, indicating partial socioeconomic overlap between the identified subgroups.

Based on the optimal AHC result, the dataset was segmented into two clusters Cluster 1 & Cluster 2 with each data point assigned to one of these groups. This stratified clustering formed the foundation for a more detailed, cluster-specific machine learning analysis in the subsequent sections, allowing for a more nuanced understanding of malaria incidence across varying socio-environmental profiles.

### Implementation of ML and H2O AutoML framework across clusters

To evaluate the performance of predictive models within different malaria risk profiles, we utilized the top ten features identified in VI. Regional and household-level variables were preserved to capture spatial and contextual heterogeneity. The whole dataset was split into two, ***DS1*** (points fall under “*Cluster 1*”) and ***DS2*** (points under “*Cluster 2*”). H2O AutoML and traditional machine learning (ML) models were trained across two datasets DS1 & DS2.

Given the presence of class imbalance in each dataset, *SMOTENC* technique was also employed to ensure more balanced and robust model performance, particularly in reducing the risk of biased predictions toward the majority class.

For traditional ML models, Bayesian Optimization was used with *10-fold cross-validation* to fine-tune hyperparameters, while H2O AutoML performed internal tuning automatically.

Further, feature importance plots from H2O AutoML provided insight into which features were most influential in driving predictions across clusters, offering valuable guidance for future medical assessments and interventions.

This section underscores not just the predictive power of AutoML frameworks like H2O, but also their practical advantages in clinical decision-making particularly in resource-constrained environments where expert tuning of complex models may not be feasible.

As shown in Table 4 result of ***DS1***, H2O GLM achieved the highest test accuracy (79.37%), recall (85.86%), and F_2_-score (83.31%), demonstrating its superior ability to identify malaria-positive cases while minimizing false-negative predictions. XGBoost ranked second, achieving a test accuracy of 75.98%, recall of 84.96%, and an F_2_-score of 82.00%, while exhibiting the smallest train–test accuracy gap (0.01 percentage points), indicating excellent model stability and generalization. SVC attained the highest precision (71.59%) but recorded the lowest recall (64.31%) and F_2_-score (65.64%), suggesting that it was less effective in detecting malaria-positive cases. The Decision Tree achieved moderate performance, with a test accuracy of 70.74%, recall of 71.24%, F1-score of 70.87%, and F_2_-score of 71.09%, whereas Logistic Regression produced a slightly lower test accuracy (68.17%) but a comparable recall (70.94%) and F_2_-score (70.16%). Overall, H2O GLM demonstrated the strongest overall performance for recall-oriented malaria screening by achieving the highest test accuracy, recall, and F_2_-score, while XGBoost exhibited the most consistent generalization performance across the training and test datasets.

**Table 4:** Performance of ML and H2O AutoML Models on Cluster 1.

Cluster	Model Name	Train Accuracy	Test Accuracy	Precision	Recall	F1-score	F2-score
**Cluster 1**	H2O GLM	77.67%	79.37%	74.46%	85.86%	79.75%	83.31%
	LR	68.68%	68.17%	67.18%	70.94%	69.01%	70.16%
	XGB	75.99%	75.98%	72.00%	84.96%	77.94%	82.00%
	DT	70.96%	70.74%	70.51%	71.24%	70.87%	71.09%
	SVC	70.70%	69.42%	71.59%	64.31%	67.75%	65.64%

**Fig 13:**
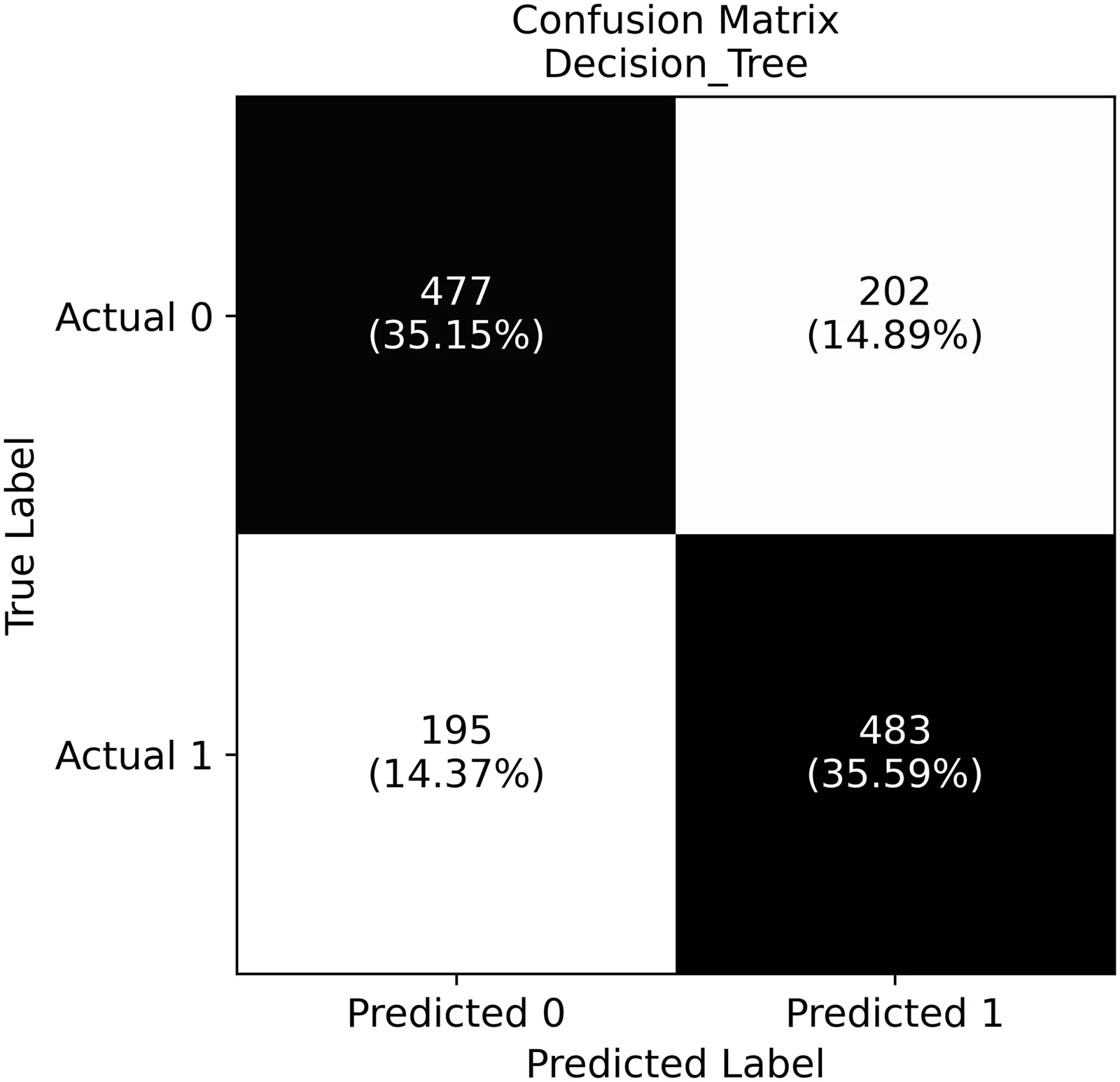
Confusion Matrix of DT of DS1.

**Fig 14:**
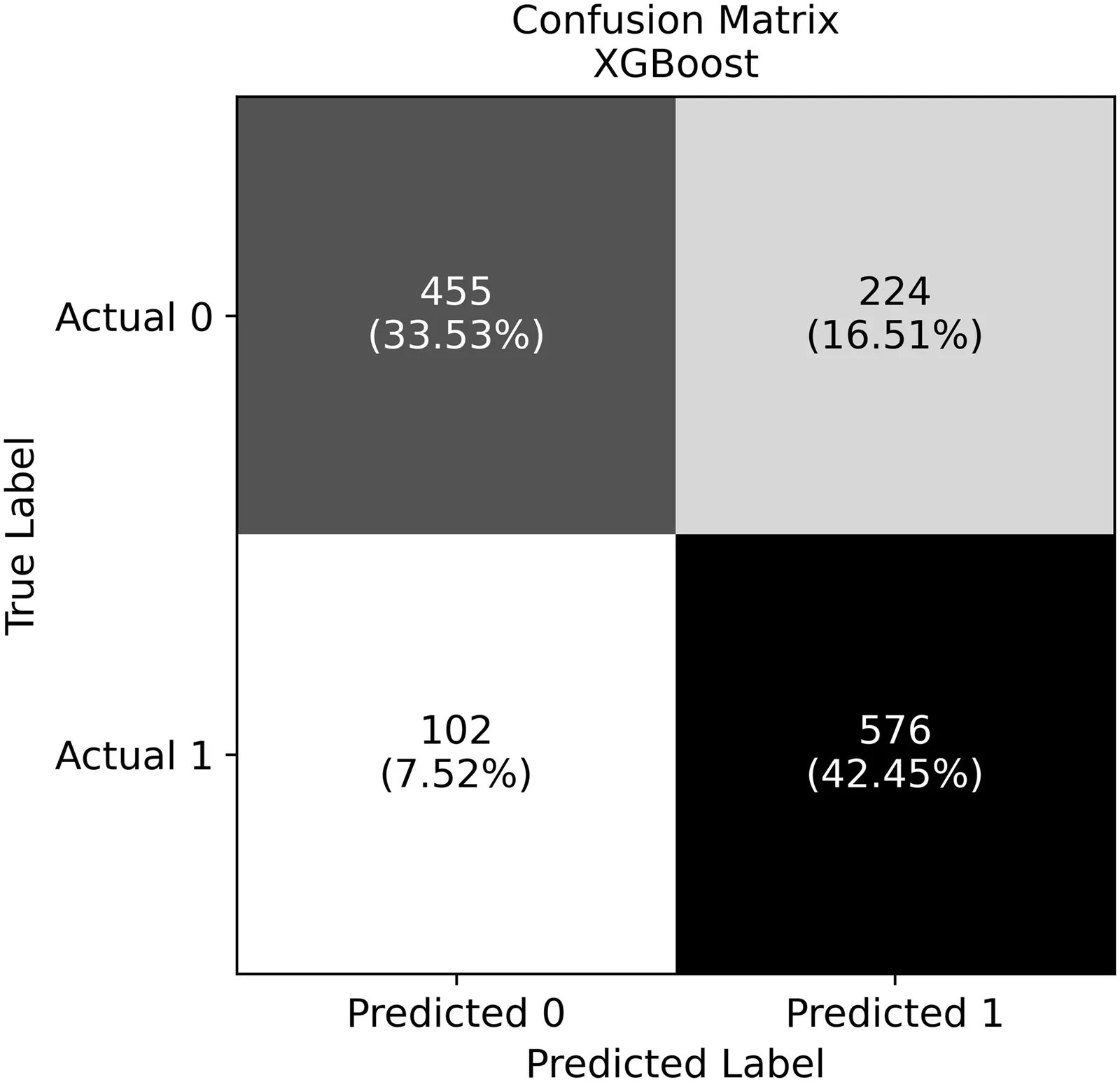
Confusion Matrix of XGB of DS1.

**Fig 15:**
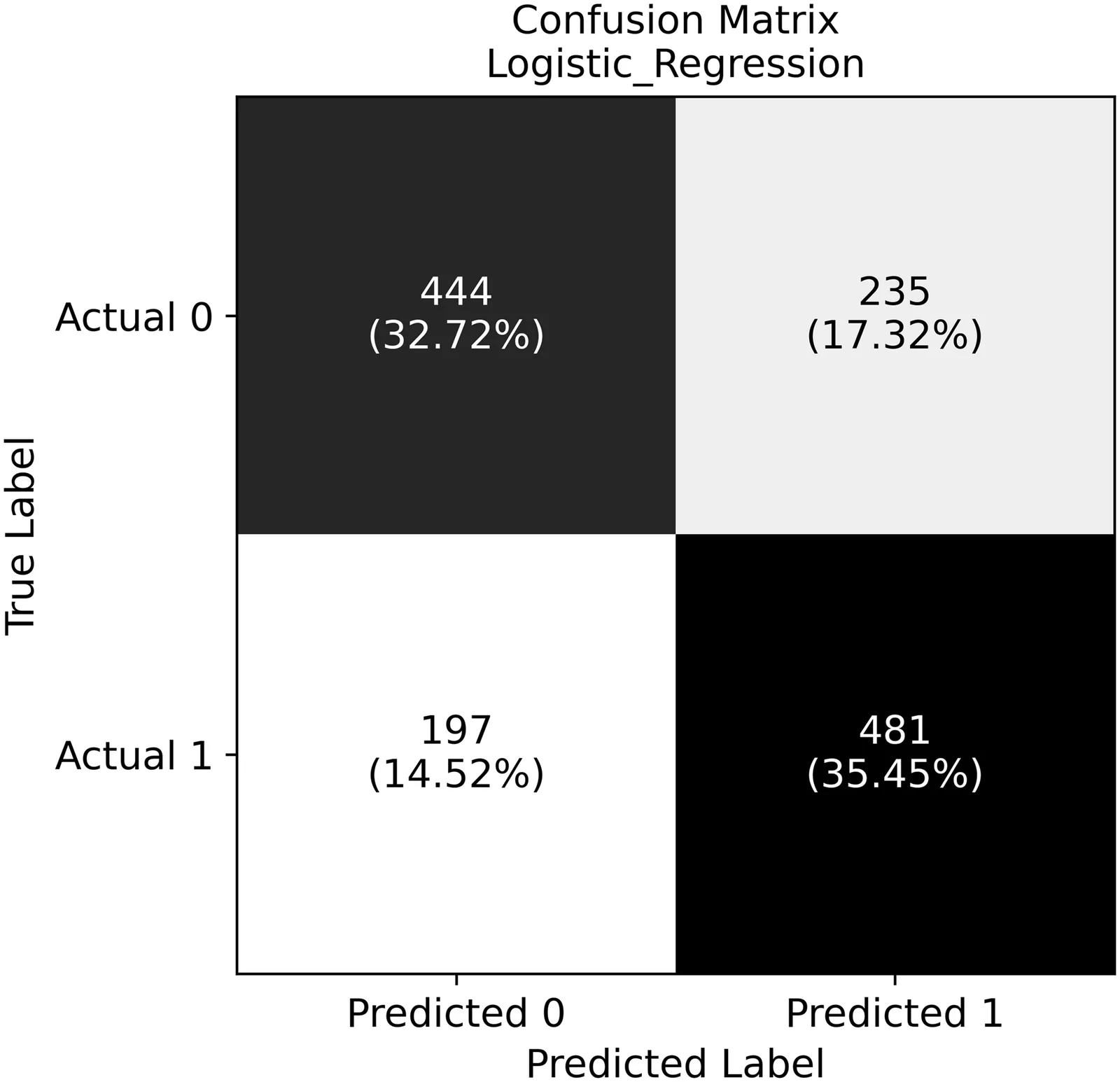
Confusion Matrix of LR of DS1.

**Fig 16:**
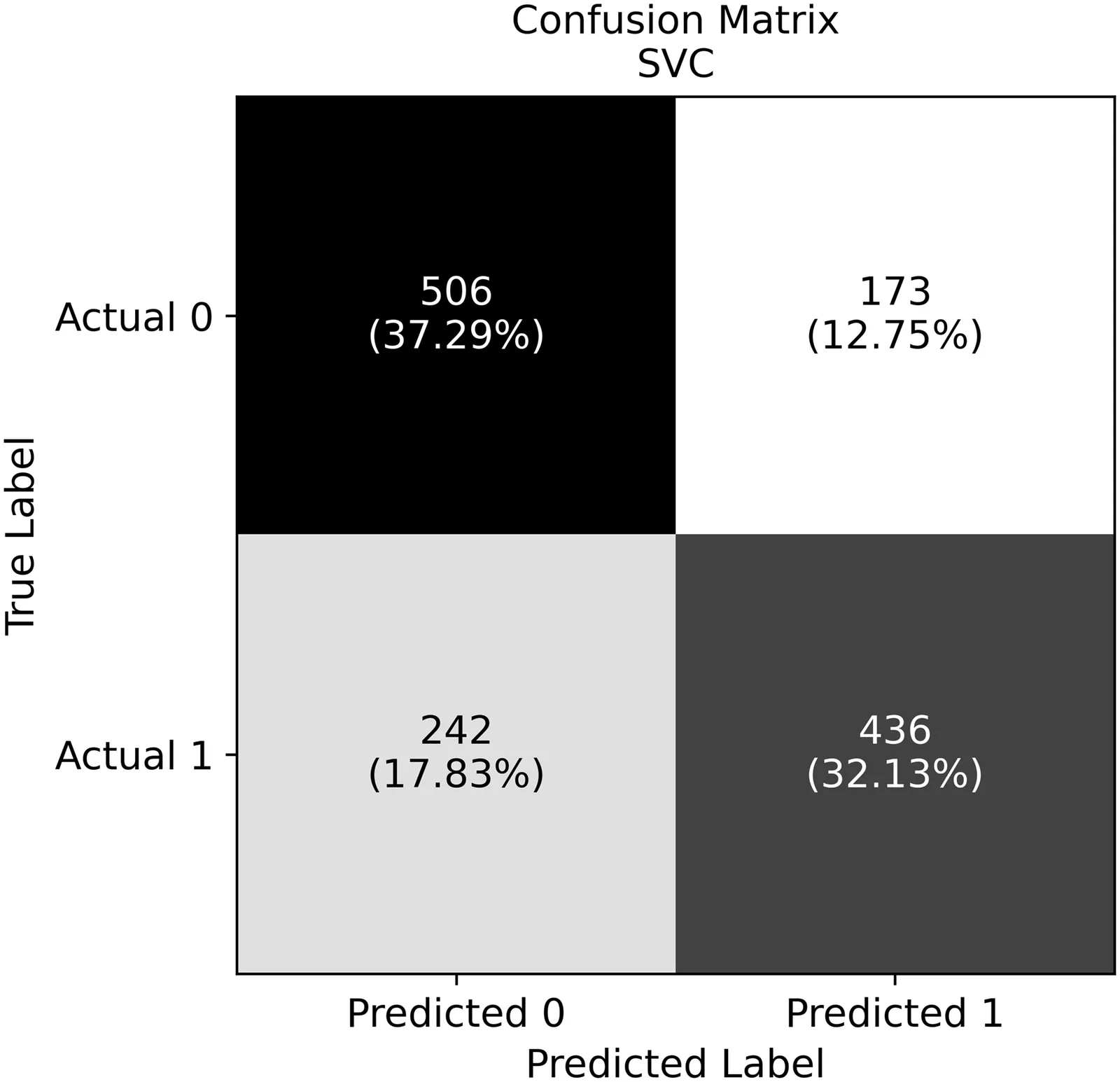
Confusion Matrix of SVC of DS1.

**Fig 17:**
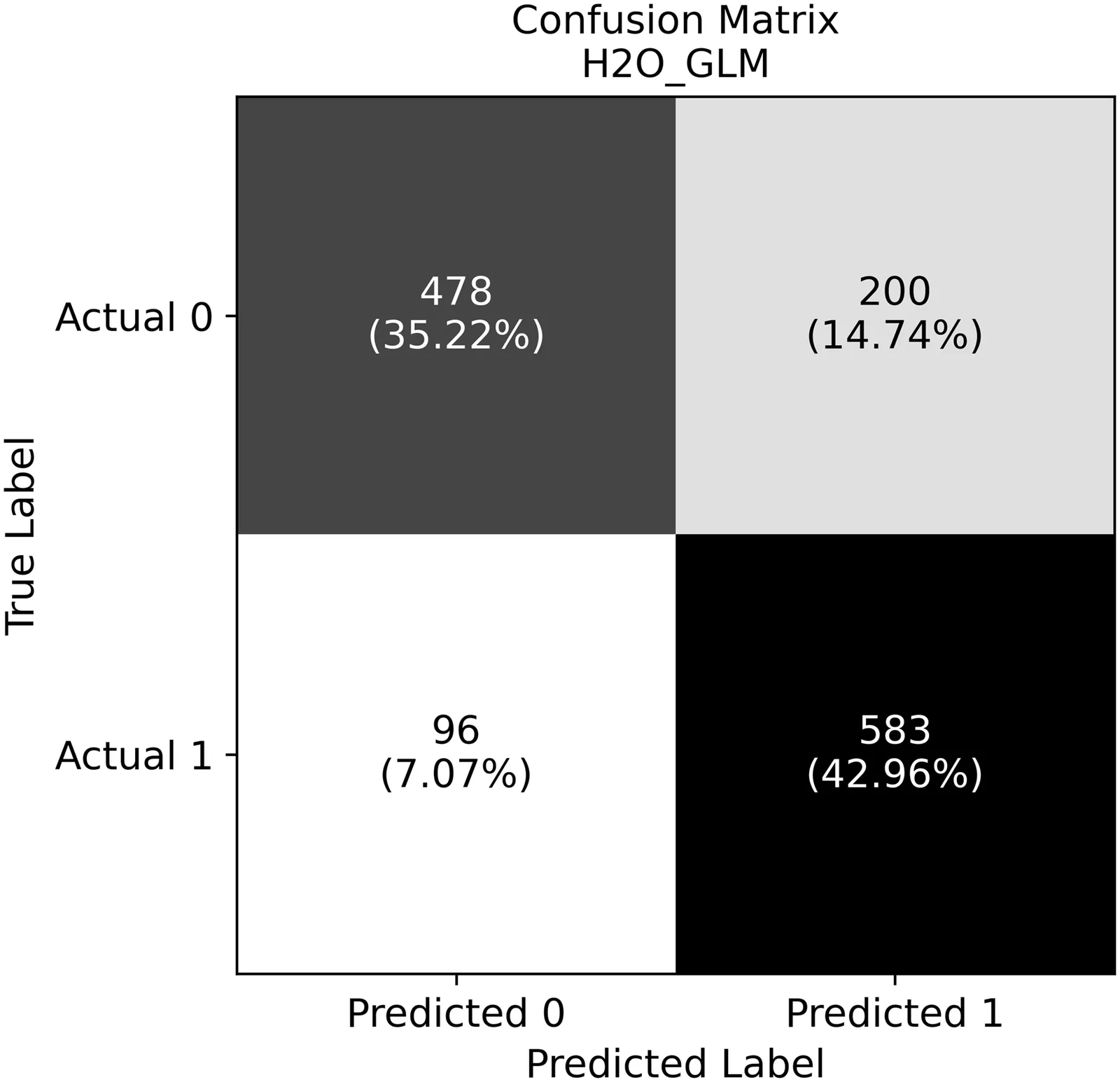
Confusion Matrix of H2O GLM of DS1.

The confusion matrices presented in Figs 13–17 demonstrate notable differences in the classification performance of the evaluated models. H2O GLM correctly identified the highest number of malaria-positive cases (583 true positives) and produced the fewest false negatives (96), indicating its superior ability to detect positive cases. XGBoost closely followed, correctly classifying 576 malaria-positive cases with only 102 false negatives, while also maintaining a balanced classification performance. Decision Tree correctly identified 483 malaria-positive and 477 malaria-negative cases, whereas SVC achieved the highest number of true negatives (506) but produced the largest number of false negatives (242), indicating lower sensitivity to malaria-positive cases. Logistic Regression exhibited comparatively weaker performance, correctly identifying 481 malaria-positive cases while generating the highest number of false positives (235).

**Fig 18:**
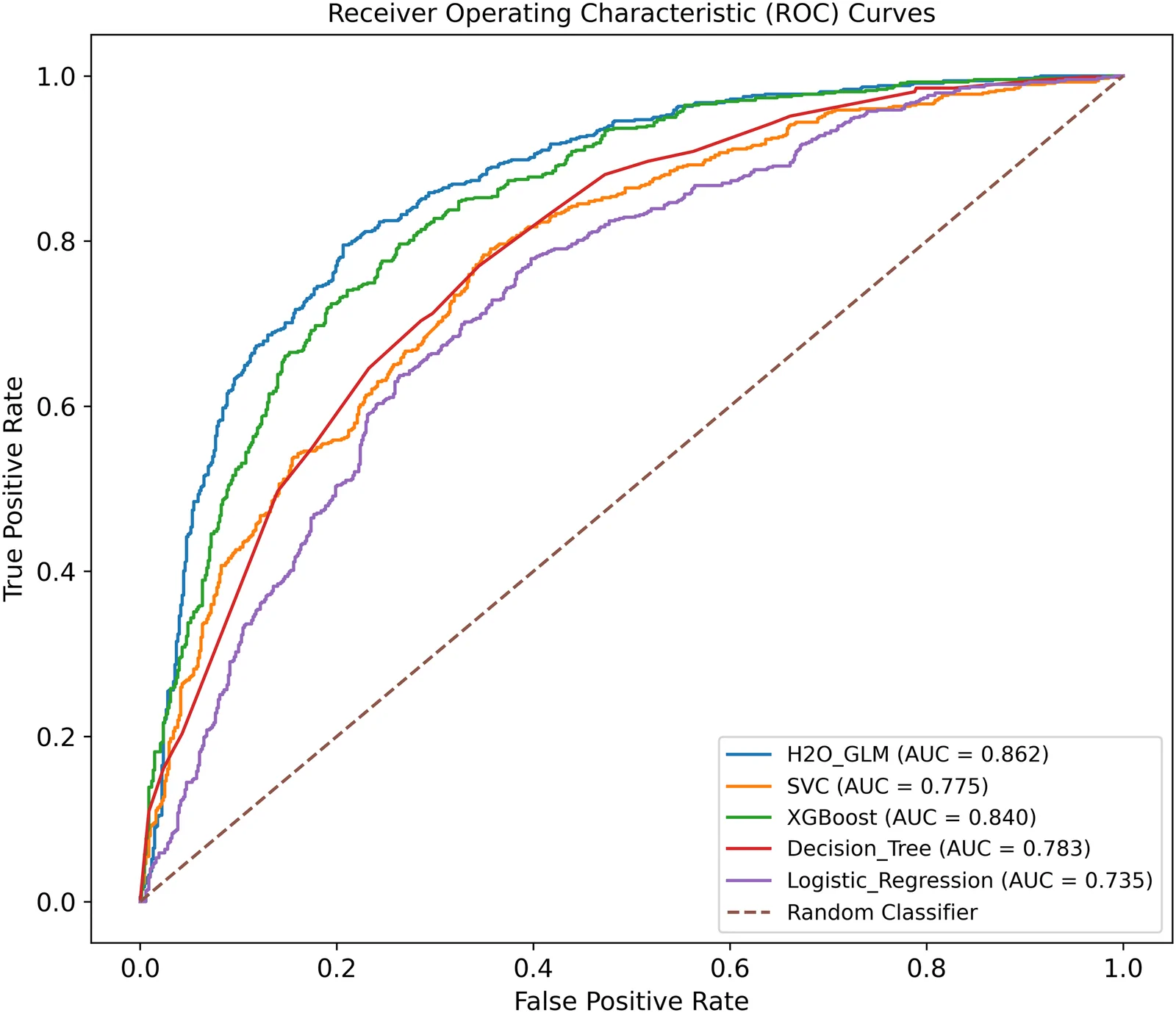
AUC-ROC of All Models of DS1.

The ROC curves (Fig 18) further highlight the discriminative ability of the models. H2O GLM achieved the highest AUC–ROC (0.862), followed by SVC (0.775), XGBoost (0.765), Logistic Regression (0.738), and Decision Tree (0.735). Overall, H2O GLM demonstrated the strongest ability to distinguish between malaria-positive and malaria-negative cases. Combined with its confusion-matrix results, H2O GLM was the most suitable model for recall-oriented malaria screening by maximizing the detection of malaria-positive cases while minimizing false-negative predictions.

**Fig 19:**
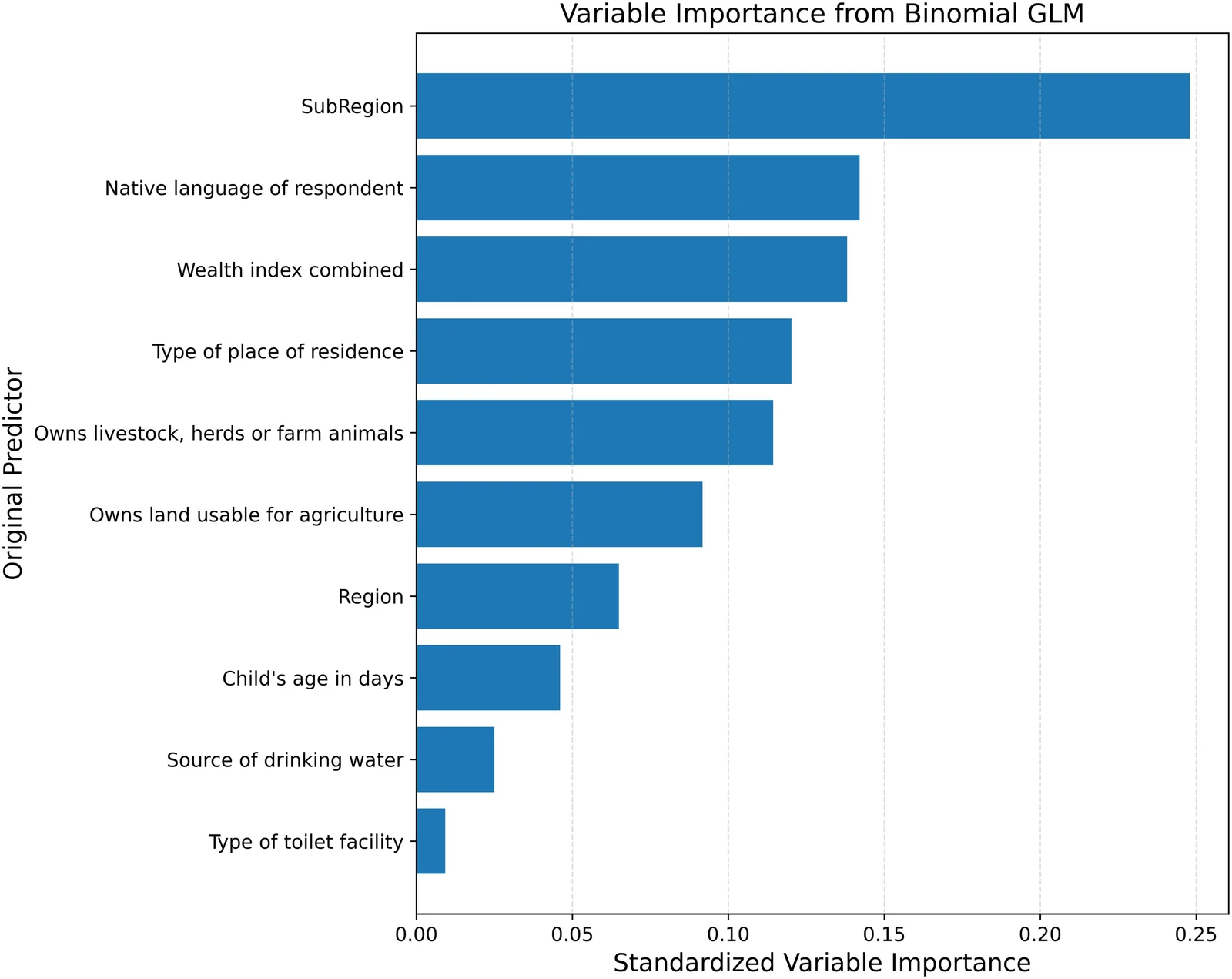
Variable Importance of GLM of DS1.

Fig 19 presents the standardized variable importance obtained from the Binomial GLM. *SubRegion* emerged as the most influential predictor, contributing substantially more to the model than the remaining variables. *Native language of respondent* and *Wealth index combined* were the second and third most important predictors, followed by *Type of place of residence*, *Ownership of livestock, herds or farm animals*, and *Ownership of land usable for agriculture*, all of which showed moderate contributions. In contrast, *Region*, *Child’s age in days*, *Source of drinking water*, and *Type of toilet facility* exhibited relatively low standardized importance, indicating a limited contribution to the model after accounting for the more influential geographic and socioeconomic factors. Overall, the results suggest that geographic location and household socioeconomic characteristics are the primary determinants influencing the model’s predictions.

As presented in Table 5 result of ***DS2***, XGBoost demonstrated the best overall predictive performance, achieving the highest recall (88.63%), F1-score (76.30%), and F_2_-score (83.25%), indicating its superior ability to identify malaria-positive cases while minimizing false-negative classifications. H2O GLM attained the highest test accuracy (68.46%) and exhibited comparable recall (85.12%) and F_2_-score (79.23%), highlighting its effectiveness in recall-oriented malaria screening. The Decision Tree provided moderate performance across all evaluation metrics, with a test accuracy of 68.65%, recall of 77.10%, and F_2_-score of 74.58%. Logistic Regression yielded lower predictive performance, achieving a test accuracy of 64.45%, recall of 68.30%, and F_2_-score of 67.26%. In contrast, SVC showed the weakest performance, recording the lowest test accuracy (62.97%), recall (53.12%), F1-score (58.92%), and F_2_-score (55.29%), indicating limited capability in identifying malaria-positive cases. Overall, the results suggest that XGBoost was the most effective model for Cluster 2, whereas H2O GLM provided competitive performance with the highest classification accuracy.

**Table 5:** Results of Traditional ML and H2O AutoML model for Cluster-2.

Cluster	Model Name	Train Accuracy	Test Accuracy	Precision	Recall	F1-score	F2-score
**Cluster 2**	H2O GLM	67.51%	68.46%	62.03%	85.12%	71.77%	79.23%
	LR	64.07%	64.45%	63.41%	68.30%	65.77%	67.26%
	XGB	76.03%	72.47%	66.98%	88.63%	76.30%	83.25%
	DT	70.05%	68.65%	65.96%	77.10%	71.10%	74.58%
	SVC	61.76%	62.97%	66.15%	53.12%	58.92%	55.29%

**Fig 20:**
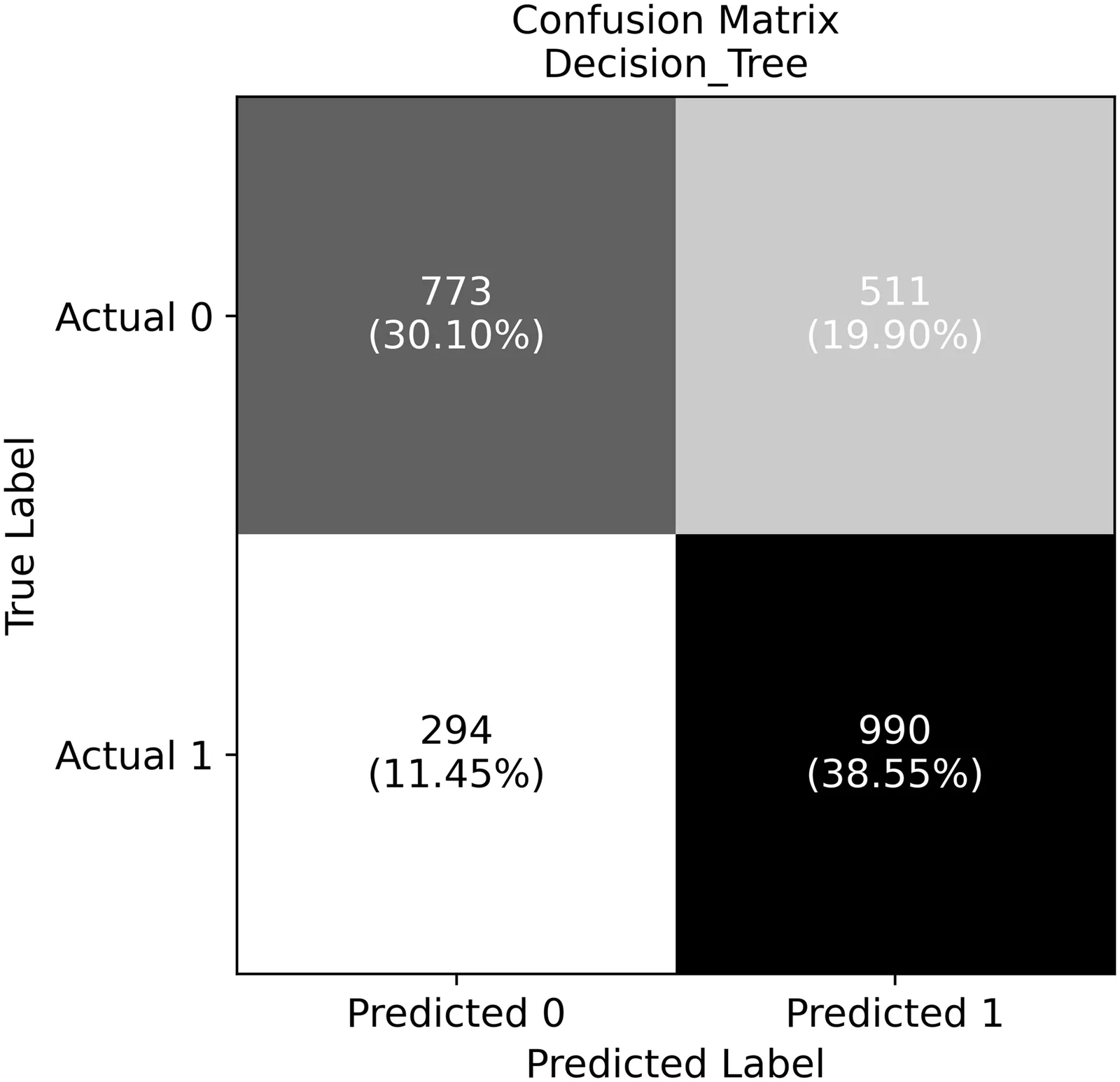
Confusion Matrix of DT of DS2.

**Fig 21:**
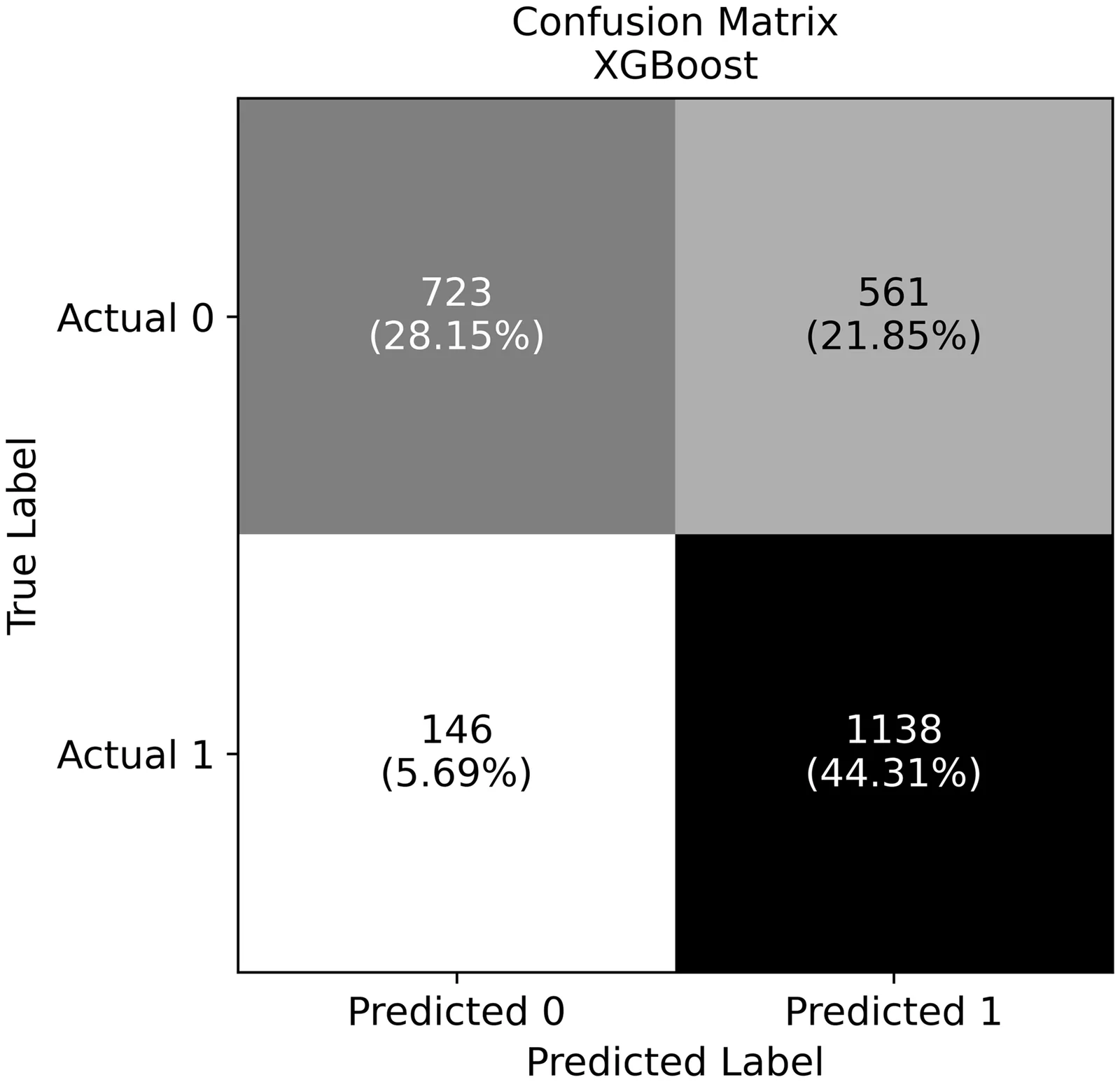
Confusion Matrix of XGB of DS2.

**Fig 22:**
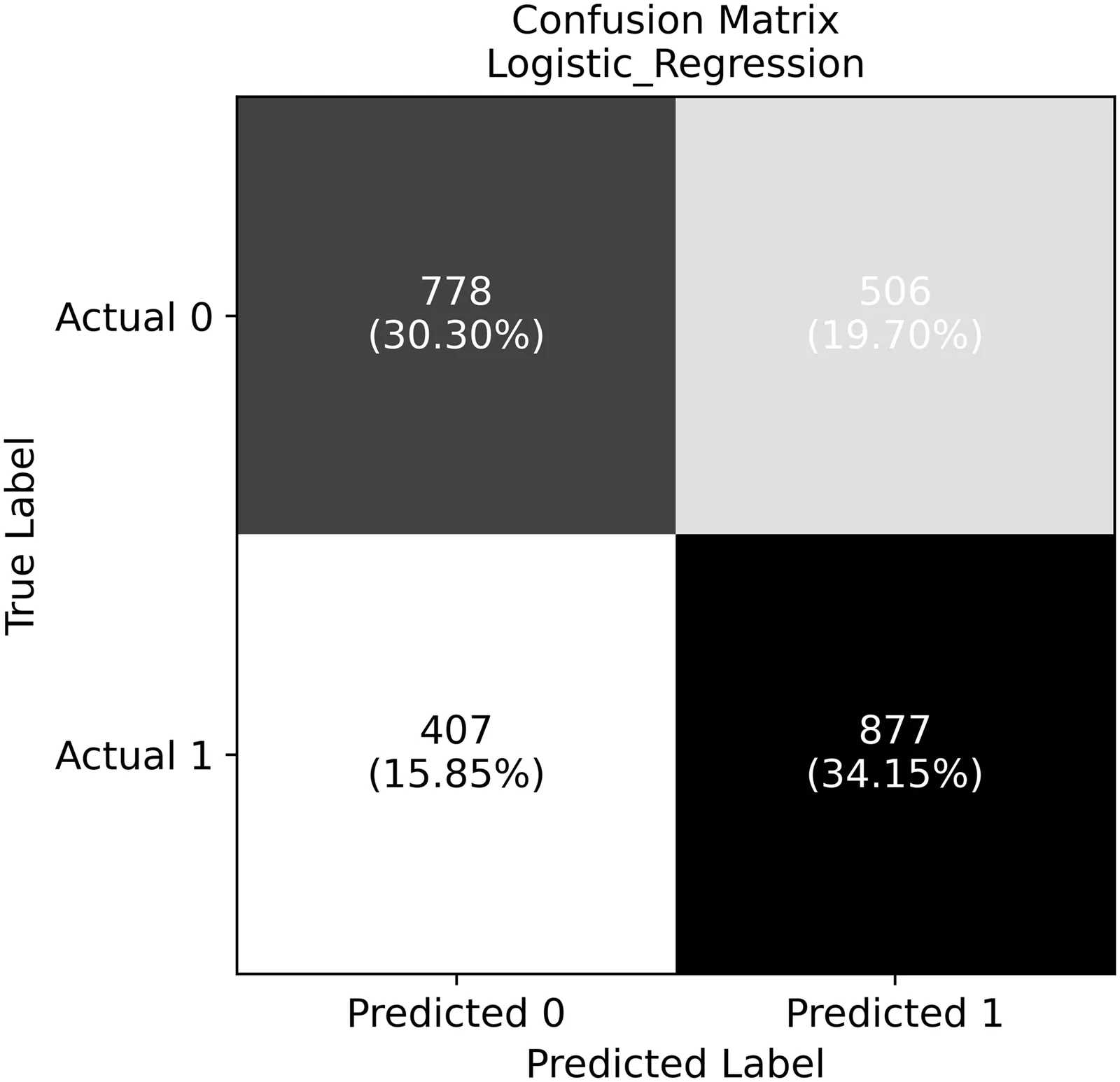
Confusion Matrix of LR of DS2.

**Fig 23:**
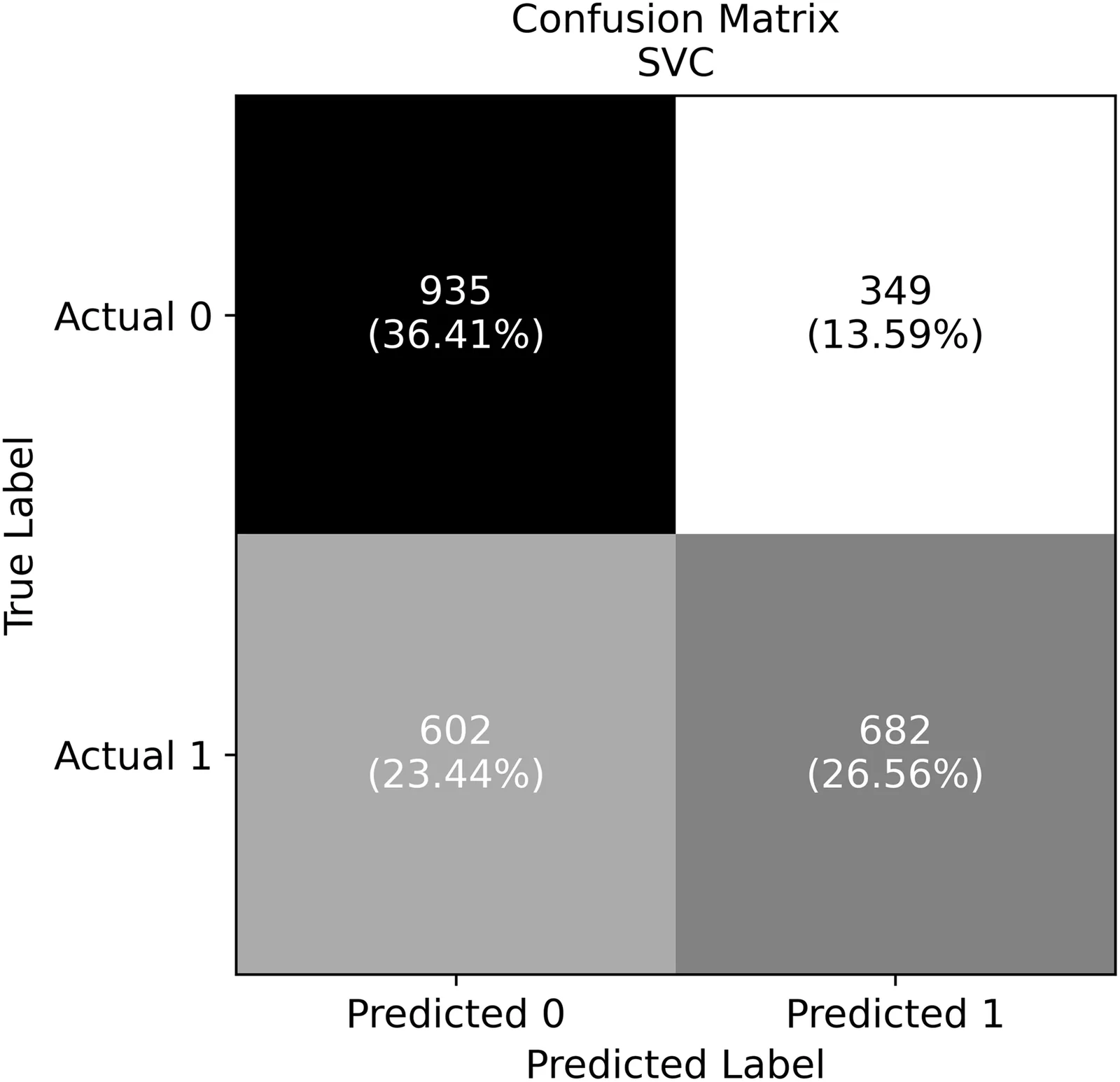
Confusion Matrix of SVC of DS2.

**Fig 24:**
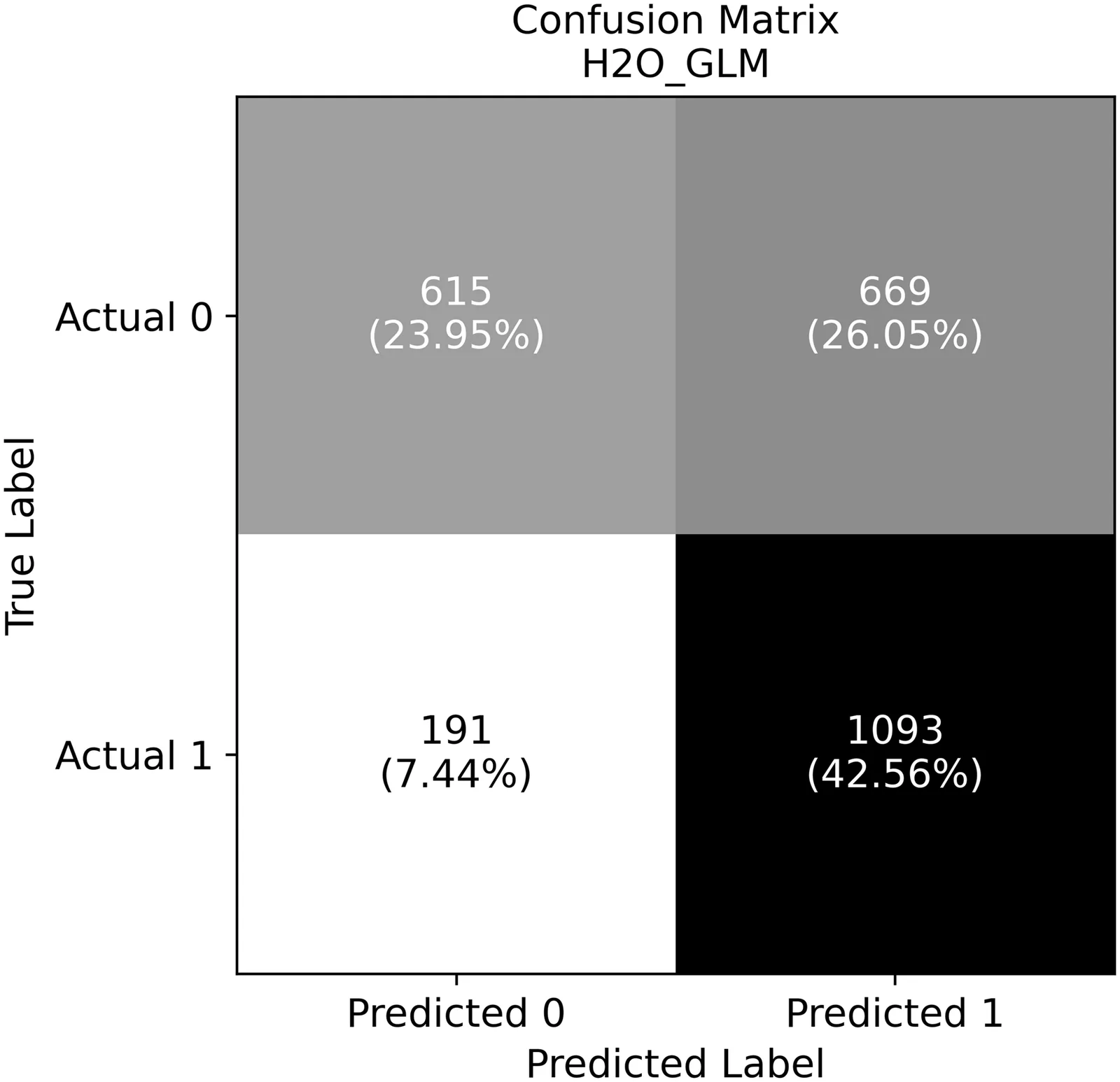
Confusion Matrix of H2O GLM of DS2.

The classification outcomes illustrated in Figs 20–24 indicate that the predictive performance varied across the evaluated models. XGBoost demonstrated the strongest capability for detecting malaria-positive cases, correctly classifying 1,138 positive samples while producing only 146 false negatives. H2O GLM also showed high sensitivity, identifying 1,093 true positives, although this was accompanied by a larger number of false positives (669). The Decision Tree provided a balanced trade-off between positive and negative classifications, with 990 true positives and 773 true negatives. In contrast, SVC achieved the highest number of true negatives (935) but failed to detect a substantial proportion of malaria-positive cases, resulting in 602 false negatives. Logistic Regression exhibited intermediate performance, correctly identifying 877 positive cases while misclassifying 407 positive instances as negative.

**Fig 25:**
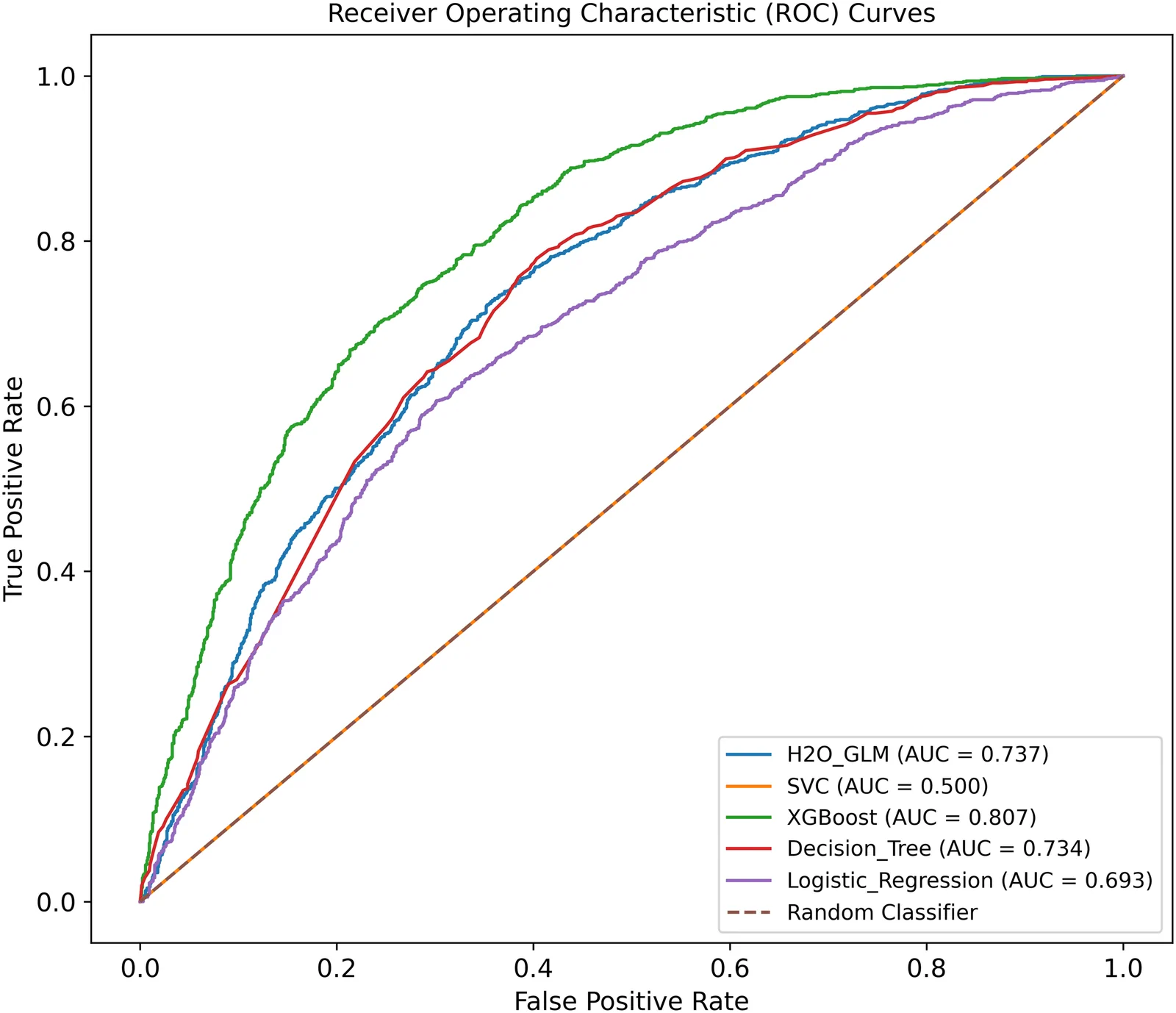
AUC-ROC of All Models of DS2.

The ROC analysis (Fig 25) further supports these findings. XGBoost achieved the largest AUC–ROC value (0.807), indicating the best overall discriminative performance, followed by Decision Tree (0.774), H2O GLM (0.737), Logistic Regression (0.693), and SVC (0.500). These results suggest that XGBoost provided the most reliable separation between malaria-positive and malaria-negative cases, whereas the discriminative ability of SVC was comparable to random classification.

**Fig 26:**
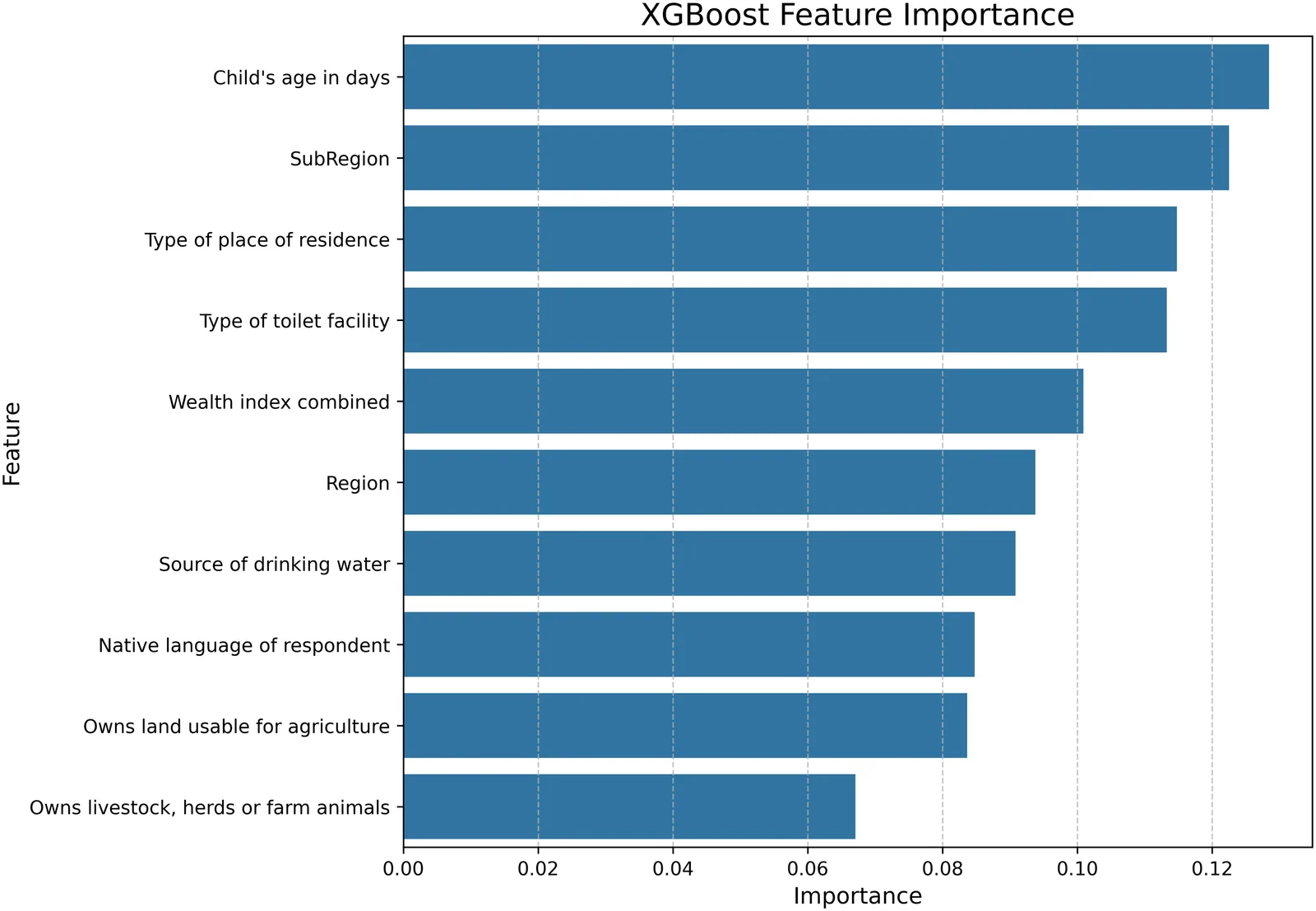
Variable Importance of XGB of DS2.

As shown in Fig 26, the XGBoost model identified *Child’s age in days* as the most important predictor for Cluster 2, followed by *SubRegion* and *Type of place of residence*. Household sanitation and socioeconomic characteristics, represented by *Type of toilet facility* and *Wealth index combined*, also demonstrated considerable predictive importance. Conversely, *Ownership of livestock, herds or farm animals* contributed the least to the model, while the remaining predictors exhibited moderate influence. These findings indicate that demographic, geographic, and household living-condition variables collectively play a central role in predicting malaria risk within Cluster 2, consistent with the superior predictive performance achieved by XGBoost for this subgroup.

## Discussion

The present study investigated the feasibility and effectiveness of the H2O AutoML framework for malaria prediction by comparing its performance with four conventional machine learning algorithms: Logistic Regression (LR), Extreme Gradient Boosting (XGBoost), Decision Tree (DT), and Support Vector Classifier (SVC). Because malaria screening prioritizes maximizing case detection while minimizing missed diagnoses, model selection primarily emphasized the F_2_-score, which assigns greater weight to recall. Test accuracy and the train–test accuracy gap were additionally considered to evaluate predictive performance and model generalization, respectively.

When considering the overall dataset, H2O GLM emerged as the most suitable model by achieving the highest F_2_-score (79.38%), demonstrating the strongest capability to identify malaria-positive individuals while minimizing false-negative predictions. Although XGBoost achieved the highest test accuracy (75.54%), its F_2_-score (78.34%) was slightly lower than that of H2O GLM. Conversely, the Decision Tree exhibited the smallest train–test accuracy gap (0.31 percentage points), indicating the most consistent generalization between the training and testing datasets. Collectively, these findings demonstrate that, although XGBoost and Decision Tree offered advantages in predictive accuracy and model stability, respectively, H2O GLM provided the most appropriate balance between sensitivity and predictive performance for recall-oriented malaria screening.

Within Cluster 1, H2O GLM similarly outperformed the competing models by achieving both the highest test accuracy (79.37%) and the highest F_2_-score (83.31%). These results indicate that H2O GLM maintained an effective balance between predictive accuracy and sensitivity within this subgroup. XGBoost achieved a comparable test accuracy (75.98%) while exhibiting the smallest train–test accuracy gap (0.01 percentage points), reflecting excellent model generalization. Nevertheless, because H2O GLM demonstrated superior recall-oriented performance, it remained the preferred model for malaria screening within Cluster 1.

In contrast, Cluster 2 exhibited a different performance profile, with XGBoost emerging as the best-performing algorithm. Specifically, XGBoost achieved both the highest F_2_-score (83.25%) and the highest test accuracy (72.47%), indicating superior discrimination and the strongest capability to identify malaria-positive individuals within this subgroup. Although Logistic Regression exhibited the smallest train–test accuracy gap (0.38 percentage points), its substantially lower F_2_-score (67.26%) suggests that improved generalization alone was insufficient to achieve effective screening performance. H2O GLM maintained high recall (85.12%) and achieved the second-highest F_2_-score (79.23%); however, XGBoost provided the most favorable balance between predictive performance and classification accuracy for Cluster 2. The superior performance of XGBoost in this subgroup may reflect the presence of more complex nonlinear relationships and feature interactions that are more effectively captured by ensemble tree-based learning than by generalized linear models.

Taken together, the findings from the overall dataset and the cluster-specific analyses demonstrate that no single algorithm consistently optimized every evaluation criterion. H2O GLM achieved the best recall-oriented performance for the overall dataset and Cluster 1, whereas XGBoost performed best in Cluster 2. These observations suggest that optimal model performance depends not only on the learning algorithm but also on the underlying characteristics and heterogeneity of the target population. Nevertheless, H2O AutoML consistently generated highly competitive models with minimal manual intervention, highlighting its practical value as an automated machine learning framework for public health applications.

Despite yielding the highest Silhouette Score among the evaluated clustering configurations, the two-cluster solution exhibited only modest cluster separation (0.3939), which was further reflected by the partial overlap observed in the MCA visualization. Therefore, the identified clusters should be interpreted as representing broad socioeconomic heterogeneity, particularly differences in wealth status, rather than well-defined, mutually exclusive population subgroups. Consequently, the cluster-specific analyses should be regarded as an exploratory assessment of predictive performance across socioeconomic subgroups, providing additional insight into population heterogeneity rather than demonstrating that clustering itself substantially improves predictive performance. It is also important to distinguish the objectives of clustering from those of prediction. Region and SubRegion were intentionally excluded from the clustering procedure to avoid defining subgroups primarily according to administrative geographic boundaries. However, these variables were retained in the predictive models because the objectives of clustering and prediction differ. While clustering was designed to reduce geographic confounding during subgroup definition, the continued importance of SubRegion as a predictor is epidemiologically expected, reflecting genuine geographic variation in malaria transmission across Nigeria.

Model robustness was further evaluated by comparing predictive performance between the training (80%) and independent held-out testing (20%) datasets to assess model generalization and the potential for overfitting. Across all datasets, the differences between training and testing accuracies remained small, indicating that the models generalized well rather than memorizing the training data. This observation was further supported by 10-fold cross-validation, in which model rankings and performance metrics remained consistent across folds, suggesting low variance and stable predictive performance. In addition, multiple regularization strategies were incorporated during model development. Conventional machine learning algorithms were optimized using Bayesian hyperparameter optimization under cross-validation constraints, whereas H2O AutoML employed internal K-fold cross-validation, early stopping, tree-depth regularization, shrinkage within gradient boosting algorithms, and stacked ensemble learning. Collectively, these strategies reduced model variance, improved generalization, and indicate that overfitting was unlikely to have substantially influenced the reported results.

Despite the encouraging predictive performance achieved in this study, external validation using independent malaria survey datasets was beyond the scope of the present work. Consequently, the generalizability of the proposed models beyond the Nigeria MIS 2021 dataset should be interpreted with appropriate caution. Future research should evaluate the framework using datasets from other malaria-endemic countries, additional survey years, or independent clinical cohorts to further assess its robustness, transportability, and applicability across diverse epidemiological settings.

Interpretability remains an important consideration for the clinical adoption of machine learning models. Although this study reports global feature importance derived from the H2O AutoML framework, these measures primarily quantify the overall contribution of individual predictors rather than explaining specific model predictions. Explainable artificial intelligence (XAI) techniques, such as SHapley Additive exPlanations (SHAP), could provide both global and instance-level interpretations by quantifying the contribution of each predictor to individual prediction outcomes. Nevertheless, the identified feature importance rankings are consistent with well-established epidemiological risk factors for malaria, indicating that the proposed models capture clinically and epidemiologically meaningful relationships. Future studies should incorporate SHAP and other advanced XAI techniques to further enhance model transparency, interpretability, and clinical trustworthiness.

Because malaria screening prioritizes maximizing case detection while minimizing false-negative predictions, the F_2_-score was selected as the primary evaluation metric. However, model evaluation was not based exclusively on the F_2_-score. Complementary performance measures, including precision, recall, F_1_-score, confusion matrices, and receiver operating characteristic (ROC) analysis, were also considered. The consistent ranking of the leading models across these complementary metrics provides additional evidence of the robustness and reliability of the proposed framework. Future research may further extend model evaluation by incorporating precision–recall curves and the area under the precision–recall curve (AUC-PR), particularly when addressing class-imbalanced datasets.

Beyond predictive performance, the H2O AutoML framework offers important practical advantages through its ability to automate the complete model development pipeline, including algorithm selection, hyperparameter optimization, model validation, and ensemble construction. Although automation does not inherently guarantee superior predictive performance, it substantially reduces manual effort while consistently identifying highly competitive models. This capability is particularly advantageous for large-scale epidemiological studies and resource-constrained research environments, where limited computational expertise or development time may otherwise restrict the practical adoption of advanced machine learning methodologies.

From a clinical implementation perspective, malaria rapid diagnostic tests (RDTs) typically provide results within 2–15 minutes, whereas confirmatory laboratory-based blood smear microscopy may require several days, particularly in resource-limited settings where laboratory infrastructure and trained personnel are limited. Consequently, the proposed AutoML framework, which utilizes routinely available demographic, socioeconomic, and household information, has the potential to function as a decision-support tool for early malaria risk assessment before confirmatory laboratory results become available. The framework may assist community health workers and primary healthcare providers in identifying children at elevated risk of malaria who should receive priority diagnostic testing, closer clinical evaluation, or earlier clinical attention, particularly in situations where rapid diagnostic tests produce false-negative results or laboratory confirmation is delayed. Such a decision-support approach could facilitate earlier identification of suspected malaria cases, improve the allocation of limited healthcare resources, strengthen malaria surveillance strategies, and support timely clinical intervention. Nevertheless, the proposed framework is intended to complement, rather than replace, standard clinical assessment and confirmatory laboratory diagnosis.

Looking ahead, the proposed framework could be integrated into a digital decision-support platform, such as a mobile or web-based application, to facilitate rapid malaria risk assessment at the point of care. The required predictor variables are routinely obtainable through standard patient interviews and household assessments without requiring laboratory investigations. If one or more predictor variables are unavailable during screening, the model output should be interpreted with caution, and the individual should undergo standard clinical assessment together with confirmatory laboratory testing. Future implementation studies should further evaluate the usability of such digital tools and investigate strategies for managing incomplete predictor information under real-world field conditions.

In summary, the findings demonstrate that H2O AutoML provides a robust, automated, and clinically relevant framework for malaria prediction using routinely available non-clinical demographic, socioeconomic, and household characteristics. Although different machine learning algorithms exhibited strengths across different population subgroups, H2O AutoML consistently produced highly competitive models with minimal manual intervention, highlighting its potential as an effective decision-support framework for early malaria risk assessment in public health settings.

## Conclusion and future work

Malaria remains a major public health challenge, particularly in sub-Saharan Africa, where timely diagnosis is essential for reducing disease burden and improving patient outcomes. This study evaluated five machine learning algorithms, including the H2O AutoML framework, for malaria prediction using the Nigeria Malaria Indicator Survey (MIS) 2021 dataset. The results demonstrate that H2O AutoML consistently produced highly competitive predictive models, achieving the best recall-oriented performance on the overall dataset and Cluster 1, while XGBoost emerged as the best-performing algorithm for Cluster 2. These findings indicate that no single algorithm is universally optimal and that model performance depends on the characteristics of the underlying population subgroup.

The cluster-specific analysis further demonstrated that spatial heterogeneity influences predictive performance, highlighting the importance of evaluating machine learning models across different population segments rather than relying solely on overall performance. Despite these variations, H2O AutoML required minimal manual intervention while delivering robust and competitive results, illustrating its practical value for automated predictive modeling in public health applications.

Future studies should investigate additional AutoML frameworks, such as Google AutoML, AutoKeras, and Auto-sklearn, using more recent and geographically diverse datasets. Incorporating explainable artificial intelligence techniques, longitudinal health records, and additional clinical variables may further improve model interpretability and predictive performance. Expanding prediction beyond binary malaria classification to include disease severity, reinfection risk, and treatment response could also enhance the clinical utility of machine learning-based decision support systems.

Overall, this study demonstrates that automated machine learning provides an effective and practical approach for malaria risk prediction. By combining strong predictive performance with reduced model development effort, AutoML has the potential to support timely clinical decision-making and strengthen malaria surveillance and screening, particularly in resource-constrained settings.

## Supporting information

S1 Table Final selected hyperparameter values by Optuna for all machine learning models trained on the 14-feature dataset, Cluster 1, and Cluster 2. (DOCX)
