# Supplementary material for: Early malaria risk screening in Nigerian minors using AutoML and cluster-based analysis of non-clinical survey data
Source: PLOS Digit Health. 2026 Sep 23;5(9):e0001736. doi: 10.1371/journal.pdig.0001736 (PMC13600517; doi:10.1371/journal.pdig.0001736)
Supplement: Supplementary file 1 [file pdig.0001736.s003.pdf]

Response Letter

Manuscript ID: PDIG-D-26-00617

Early Malaria Risk Screening in Nigerian Minors Using AutoML and Cluster-Based Analysis of Non-Clinical Survey Data

PLOS Digital Health

Response to the Academic Editor

We sincerely thank Dr. Cleva Villanueva for the careful evaluation of our manuscript and for the constructive comments and recommendations. We have carefully considered each point and substantially revised the manuscript accordingly. Detailed responses to each comment are provided below.

Comment 1. Potential Target Leakage and Consistency with the Study Objective

The manuscript presents the proposed framework as a non-clinical malaria screening approach based on sociodemographic and household characteristics. However, some of the most important predictors included in the models appear to be clinical or laboratory-derived variables, including malaria rapid diagnostic test results and Plasmodium falciparum status. The authors should carefully reconsider the inclusion of these variables, provide a strong justification for their use, and discuss their implications for the intended use of the model. Ideally, the authors should evaluate model performance after removing clinical and laboratory variables to determine whether the framework can truly function as a non-clinical screening tool.

We thank the Academic Editor for this important observation. In response, we completely removed the two clinical/laboratory-derived variables ("Result of malaria rapid test" and "Presence of species: falciparum") from the predictive modeling framework to eliminate potential target leakage and ensure consistency with the study objective of developing a non-clinical malaria risk screening tool. Following their removal, all machine learning models, including the H2O AutoML framework and conventional machine learning algorithms, were retrained using only non-clinical demographic, household, and socioeconomic predictors. Consequently, all performance metrics, tables, figures, the Abstract, Author Summary, Results, Discussion, and Conclusion were updated to reflect the revised analyses. The revised manuscript now consistently presents the proposed framework as a non-clinical decision-support approach for early malaria risk assessment.

Comment 2. Survey Design and Representativeness

Additional information is needed regarding the handling of the Nigeria Malaria Indicator Survey design. It is currently unclear whether sampling weights, clustering, and stratification were incorporated appropriately into the analyses. Given the complex survey design of the MIS dataset, the authors should provide a detailed explanation of how these elements were handled and discuss any implications for the validity and generalizability of the findings.

*We appreciate this valuable suggestion. A dedicated **Survey Design** subsection has been added to the Methods. The revised manuscript now explains that the Nigeria Malaria Indicator Survey (MIS) 2021 employed a nationally representative multistage stratified cluster sampling design. We further clarify that the objective of the present study was predictive machine learning rather than estimation of nationally representative epidemiological parameters. Therefore, survey sampling weights, strata, and primary sampling unit (PSU) information were not incorporated into model development. Instead, stratified train–test splitting based on the malaria outcome variable and nested cross-validation were employed to ensure balanced model development and robust performance evaluation. The implications of this methodological choice for model interpretation and generalizability are also discussed.*

Comment 3. Missing Figures and Reproducibility

Several figures referenced in the manuscript were not available for review. These include confusion matrices, ROC curves, feature importance plots, clustering visualizations, and silhouette analyses. These figures are essential for evaluating model performance, class separation, clustering validity, and interpretability. The authors should ensure that all referenced figures are included in the revised submission.

We thank the Academic Editor for identifying this omission. All figures referenced in the manuscript have now been included in the revised submission. These include the confusion matrices, ROC curves, variable importance plot, multiple correspondence analysis (MCA) visualization, silhouette score analysis, and all other referenced figures. Their inclusion improves the transparency, interpretability, and reproducibility of the proposed framework.

Comment 4. Clinical Translation and Practical Implementation

The manuscript would benefit from a more detailed discussion regarding how the proposed framework could be implemented in real-world settings, including how community health workers would use the model, how predictor variables would be collected, how missing information would be handled, and whether a digital implementation strategy is envisioned.

*We appreciate this constructive recommendation. The Discussion has been substantially expanded to clarify the potential practical implementation of the proposed framework. We now describe how routinely available demographic, household, and socioeconomic variables can be collected through standard patient interviews and household assessments, enabling community health workers and primary healthcare providers to use the framework as an early decision-support tool in resource-limited settings. We further discuss that **future implementation** of the proposed framework could involve its integration into a mobile- or web-based digital decision-support platform to facilitate rapid malaria risk assessment at the point of care. In addition, we clarify that when one or more predictor variables are unavailable, the model output should be interpreted with caution, and individuals should undergo standard clinical assessment together with confirmatory laboratory testing. The revised Discussion also emphasizes that the proposed framework is intended to complement, rather than replace, established clinical diagnostic practices.*

Comment 5. External Validation

The lack of external validation should be discussed more explicitly as an important limitation.

We thank the Academic Editor for this important suggestion. The Discussion has been revised to explicitly acknowledge the absence of external validation as a limitation of the present study. We now state that the proposed framework was evaluated using the Nigeria MIS 2021 dataset only and that future studies should validate the models using independent datasets from other malaria-endemic countries, additional survey years, or external clinical cohorts before large-scale implementation.

Additional Methodological Clarifications

Following the Academic Editor's recommendations, several additional methodological improvements have been incorporated throughout the manuscript. These include: (i) clarification of the feature selection and missing-data handling procedures, including the progression from 43 candidate variables to 15 statistically evaluated variables and the final 13 predictors used for model development; (ii) additional details describing the agglomerative hierarchical clustering procedure, including the use of Gower distance, average linkage, and silhouette analysis; (iii) complete reporting of the H2O AutoML configuration and training settings to improve reproducibility; (iv) expanded interpretation of the revised two-cluster solution, including its socioeconomic characteristics. Specifically, the revised manuscript explains that Cluster 0 contains a higher proportion of individuals from the Richest and Richer wealth categories, whereas Cluster 1 contains more individuals from the Poorest and Poorer categories, with partial overlap across the intermediate Poor category, together with a discussion of the implications for subgroup-specific model performance;; (v) verification and updating of all reported performance metrics, tables, and figures following model retraining; and (vi) revision of the Discussion to incorporate additional relevant literature, reduce excessive methodological descriptions of machine learning algorithms, provide a more balanced interpretation of artificial intelligence, and discuss the limitations associated with survey-derived predictors for individual-level prediction.

Comments from Reviewer #1

We sincerely thank the reviewer for the careful evaluation of our manuscript and for the valuable comment regarding the treatment of the Nigeria Malaria Indicator Survey (MIS) design. We have carefully considered this recommendation and revised the manuscript accordingly.

Comment 1. Survey design

The handling of Nigeria MIS survey design seems a bit insufficient because it gave me an impression that all observations were treated as independent and unweighted.

We thank the reviewer for this important observation. To address this concern, we have added a dedicated ***Survey Design*** *subsection to the Methods. The revised manuscript now explains that the Nigeria Malaria Indicator Survey (MIS) 2021 employed a nationally representative multistage stratified cluster sampling design. We further clarify that the primary objective of the present study was the development and evaluation of predictive machine learning models rather than the estimation of nationally representative epidemiological parameters. Consequently, survey sampling weights, stratification, and primary sampling unit (PSU) information were not incorporated into model development. Instead, stratified train–test splitting based on the malaria outcome variable and nested cross-validation were employed to ensure balanced model development and robust performance evaluation. We also discuss the implications of this methodological choice for the interpretation and generalizability of the findings.*

Comments from Reviewer #2

We sincerely thank the reviewer for the careful evaluation of our manuscript and for the constructive comments and suggestions. We appreciate the recognition of the clinical importance of the study, the comprehensive methodology, the appropriate use of the F_2-score, and the spatial analysis. We have carefully addressed each comment, and the manuscript has been substantially revised accordingly. Our detailed responses are provided below.

Comment 1. The authors may wish to mention other relevant studies in the discussion part.

*We thank the reviewer for this thoughtful suggestion. We carefully considered incorporating additional comparative studies into the Discussion. However, the primary objective of this study was to develop a **non-clinical malaria risk screening framework** based exclusively on demographic, household, and socioeconomic variables. To the best of our knowledge, relatively few published studies have adopted a comparable non-clinical predictive framework using nationally representative survey data. Because of these methodological differences, direct comparison with studies based on clinical, laboratory, or imaging data would not provide a meaningful evaluation of our findings. Therefore, we focused the Discussion on interpreting the results of the proposed framework, its practical implications, strengths, and limitations within the context of non-clinical malaria risk screening.*

Comment 2. Authors should mention why all partially missing features were removed. How many features were removed, and which specific features were excluded? Were they clinically or epidemiologically important?

*We appreciate this important comment. The preprocessing and feature-selection procedures have been clarified in the revised Methods section. The manuscript now explains that observations with missing malaria outcome values were removed before analysis. Variables with substantial missingness or insufficient data quality were excluded during preprocessing because they could not support reliable statistical analysis or model development. Initially, **43 candidate variables** were selected from the Nigeria MIS 2021 dataset based on their potential relevance to malaria prediction. Following preprocessing and data quality assessment, **15 variables** were retained for statistical evaluation. Chi-square and Kruskal–Wallis tests subsequently identified two variables, **"Has mosquito bed net for sleeping"** and **"Sex of the Child,"** as not significantly associated with the malaria outcome ($p > 0.05$). These variables were excluded, resulting in a final set of **13 predictor variables** used for clustering and machine learning model development. Regarding the variables excluded during preprocessing because of extensive missingness, statistical association with the outcome could not be reliably evaluated due to insufficient available observations. Consequently, we could not determine whether these variables were clinically or epidemiologically important within the context of the present dataset, and they were excluded solely on data quality grounds to ensure robust and reliable model development.*

Comment 3. Including positive RDTs as a feature can lead to potential leakage. If the objective is to develop an early screening framework based primarily on household and socio-demographic factors, the authors should consider excluding RDT-related variables from the predictive modelling pipeline or provide stronger justification for their inclusion.

*We thank the reviewer for this important observation. In response, we completely removed the clinical and laboratory-derived variables **"Result of malaria rapid test"** and **"Presence of species: falciparum"** from the predictive modeling framework to eliminate potential target leakage and ensure consistency with the objective of developing a non-clinical malaria risk screening tool. Following their removal, all machine learning models, including the H2O AutoML framework and conventional machine learning algorithms, were retrained using only demographic, household, and socioeconomic predictors. Consequently, all performance metrics, tables, figures, and related sections of the manuscript, including the Abstract, Author Summary, Results, Discussion, and Conclusion, were comprehensively updated to reflect the revised analyses. As expected, the predictive performance of all models decreased compared with the original submission, resulting in more moderate and realistic performance estimates after eliminating information leakage. In addition, the clustering analysis was repeated using the revised feature set, and the optimal clustering solution changed from **three clusters to two clusters**, demonstrating that the removal of the leakage-related variables also altered the underlying population structure captured by the clustering algorithm. These revisions ensure that the proposed framework is fully aligned with its intended purpose as a **non-clinical early malaria risk screening tool**.*

Comment 4. Authors may wish to clearly mention external validation in the limitation part.

We appreciate this valuable suggestion. The Discussion has been revised to explicitly acknowledge the absence of external validation as an important limitation of the present study. We now state that the proposed framework was evaluated using only the Nigeria MIS 2021 dataset and that future studies should validate the models using independent datasets from other malaria-endemic countries, additional survey years, or external clinical cohorts before clinical or public health deployment.

Comment 5. Cluster interpretation should be mentioned clearly. What distinguishes clusters 1, 2, and 3?

*We thank the reviewer for this valuable comment. Following the complete redevelopment of the predictive framework after removing the clinical and laboratory-derived variables, the clustering analysis was repeated. The revised analysis identified an optimal **two-cluster** solution, rather than the three clusters reported in the original submission, based on the highest Silhouette Score (0.3939). The manuscript has been updated to provide a more comprehensive description of the clustering methodology, including the use of Gower distance, average linkage, and multiple correspondence analysis (MCA) for cluster visualization. In addition, the characteristics of the identified clusters are now described more clearly. Specifically, **Cluster 0 contains a higher proportion of individuals in the Richest and Richer wealth categories, whereas Cluster 1 comprises a higher proportion of individuals in the Poorest and Poorer wealth categories. The intermediate Poor wealth category is represented similarly across both clusters, indicating partial socioeconomic overlap between the identified subgroups.** These findings, together with the MCA visualization, suggest that the clusters capture meaningful latent socioeconomic heterogeneity, enabling subgroup-specific model development and demonstrating that different machine learning algorithms perform optimally across different population subgroups.*

Comment 6. Some tables appear to contain identical performance values across different models. Please ensure they are reported correctly.

We appreciate the reviewer for identifying this issue. Following the removal of the clinical and laboratory-derived variables, all machine learning models were retrained and every performance metric was recalculated. Accordingly, all tables, figures, and reported performance measures have been thoroughly verified and updated. The revised manuscript now reports the corrected performance metrics derived from the retrained models, ensuring complete consistency throughout the manuscript.

Comment 7. Some parts of the manuscript overly discussed AI models. These explanations may be unnecessarily detailed for the target readership and could be shortened to improve readability and maintain focus on the study's methodological contribution and findings.

We thank the reviewer for this valuable suggestion. The methodological descriptions of the machine learning algorithms were carefully reviewed and streamlined where appropriate to improve readability while retaining the information necessary to understand the modeling approach. Redundant textbook-style explanations were condensed for the Logistic Regression, Decision Tree, and Support Vector Classifier algorithms. However, the descriptions of the XGBoost algorithm and the Bayesian optimization procedure were intentionally retained because they are closely linked to the mathematical formulations and optimization strategy presented in the manuscript. Further reduction of these sections would compromise the clarity and interpretation of the corresponding equations and methodology. Overall, the revisions place greater emphasis on the study design, methodological contribution, model evaluation, and practical implications of the proposed framework while maintaining sufficient technical detail to ensure clarity and reproducibility.

Comments from Reviewer #3

We sincerely thank the reviewer for the thorough evaluation of our manuscript and for the insightful comments and recommendations. We appreciate the reviewer's careful assessment, which has substantially improved the methodological rigor, transparency, and practical relevance of our study. Detailed responses to each comment are provided below.

Comment 1. Target Contamination and Data Leakage (The 15-Feature Model)

The manuscript frames this work as a "non-clinical early malaria risk screening framework"... The authors must strictly isolate the non-clinical boundary... The manuscript frames this work as a "non-clinical early malaria risk screening framework leveraging household and socio-economic data" to step in when rapid diagnostic tests (RDTs) or microscopy are delayed or unavailable. However, the 15-feature model includes "Presence of species: falciparum (Pf)" and "Result of malaria rapid test" as heavily weighted baseline features.

- "Presence of species: falciparum" requires laboratory microscopy or PCR to be determined. If a clinician already possesses parasitological laboratory confirmation of the species, a non-clinical machine learning screening model is redundant.

- Incorporating the "Result of malaria rapid test" to predict the final blood smear means the model is using a clinical diagnostic proxy of the target. This directly contradicts the premise that the tool operates when clinical testing is unavailable.

- Action Required: The authors must strictly isolate the non-clinical boundary. All clinical/laboratory variables must be completely removed from both the national and cluster-specific screening models. The authors must retrain the pipelines purely on true socio-demographic, environmental, and household features, and transparently discuss the resulting shifts in performance metrics

*We sincerely thank the reviewer for this critical observation. We fully agree that including the variables **"Result of malaria rapid test"** and **"Presence of species: falciparum"** was inconsistent with the intended purpose of a non-clinical malaria risk screening framework and introduced the potential for target leakage. In response, both clinical and laboratory-derived variables have been completely removed from the predictive modeling pipeline. All machine learning models, including the H2O AutoML framework and conventional machine learning algorithms, were retrained using only demographic, household, and socioeconomic predictors.*

*Consequently, all performance metrics, tables, figures, and related sections of the manuscript, including the Abstract, Author Summary, Results, Discussion, and Conclusion, were comprehensively updated. As expected, the predictive performance of all models decreased compared with the original submission, yielding more moderate and realistic performance estimates after eliminating information leakage. Furthermore, the clustering analysis was repeated using the revised predictor set, and the optimal clustering solution changed from **three clusters to two clusters**, indicating that the removal of the leakage-related variables also altered the underlying population structure identified by the clustering algorithm. These revisions ensure that the proposed framework is fully consistent with its intended role as a **non-clinical early malaria risk screening tool**.*

Comment 2. Missing Evaluation Figures

The main text frequently references essential visual aids that are entirely absent from the provided manuscript files:

- Page 25 references Figures 3 through 7 (Confusion Matrices) and Figure 8 (AUC-ROC Curves).

- Page 25 references Figure 9 (Variable Importance of Gradient Boosting).

- Page 28 references Figure 10 (2D Projection via MCA) and Figure 11 (Silhouette Profile).

- Action Required: The authors must embed or provide these figures. Reviewers cannot validate classification boundaries, class-imbalance impacts, or clustering thresholds without inspecting these plots.

We thank the reviewer for identifying this omission. All previously missing figures have now been included in the revised manuscript. These include the confusion matrices, ROC curves, variable importance plot, silhouette score analysis, and the two-dimensional multiple correspondence analysis (MCA) projection of the identified clusters. Their inclusion improves the transparency, interpretability, and reproducibility of the proposed framework.

Comment 3. Translational and Operational Feasibility

The discussion section should address how an macro-level national household survey model translates to an active point-of-care digital health intervention. Features such as "Wealth index combined", "Type of toilet facility", and "Time to get to water source" are structural, seasonal, or aggregate indicators.

- Action Required: Clarify how a community health worker in a low-resource setting would operationalize this score during an acute clinical presentation. Is there an intended mobile interface? How will missing data fields at the individual level be dynamically accommodated by the H2O Stacked Ensemble?

We appreciate this valuable recommendation. The Discussion has been substantially expanded to clarify the potential implementation of the proposed framework in real-world settings. We now explain how routinely available demographic, household, and socioeconomic variables can be collected through standard patient interviews and household assessments, allowing community health workers and primary healthcare providers to use the framework as an early decision-support tool in resource-limited settings. We also discuss the potential integration of the framework into mobile or web-based digital decision-support platforms. Furthermore, we clarify that the framework is intended to support, rather than replace, clinical assessment and confirmatory laboratory testing. When one or more predictor variables are unavailable, model predictions should be interpreted cautiously and clinical judgment should take precedence.

Comment 4. Clustering Reproducibility

The authors state that an optimal Silhouette Score of 0.510 was achieved with three clusters using Agglomerative Hierarchical Clustering. To ensure complete technical reproducibility, please explicitly report the distance metric (e.g., Euclidean) and the linkage criteria (e.g., Ward’s, Complete) utilized.

We thank the reviewer for this helpful suggestion. The clustering methodology has been expanded in the revised Methods section. The manuscript now explicitly states that Agglomerative Hierarchical Clustering was performed using ***Gower distance*** *to accommodate mixed categorical and numerical variables, followed by hierarchical clustering using the* ***average linkage*** *criterion. The optimal number of clusters was determined by evaluating* ***Silhouette Scores*** *for cluster solutions ranging from* ***2 to 10****, with the highest score (****0.3939****) obtained for the two-cluster solution.*

Comment 5. Hyperparameter Transparency in AutoML Class Imbalance

The manuscript notes that traditional ML models were under-sampled to handle class imbalance, while H2O AutoML was left untouched because it "handles imbalanced data internally." By default, tree-based algorithms inside H2O maximize standard accuracy. Please state the exact parameters passed to the H2O pipeline (e.g., balance_classes=True, or explicit setting of stopping_metric="F2") to clarify how the pipeline prioritized recall over majority-class dominance.

We appreciate this important comment. The revised manuscript now reports the complete H2O AutoML configuration, including the values of max_models = 10, seed = 123, nfolds = 10, sort_metric = AUCPR, stopping_metric = AUCPR, stopping_rounds = 3, stopping_tolerance = 0.001, and the excluded algorithms = [Deep Learning and Stacked Ensemble]. We also clarify that class imbalance was addressed by applying ***SMOTENC exclusively to the training dataset before model development****. Because the H2O AutoML framework was trained using this already balanced training dataset, the balance_classes parameter was not enabled. This approach ensured that all machine learning models, including H2O AutoML, were trained and evaluated under identical class distributions, enabling a fair comparison of predictive performance.*

Comment 6. Citation Mismatch in Table 1

In Section 1 (Table 1), recent literature benchmarks are hardcoded as static text brackets (e.g., [16], [17]). Ensure these entries are properly linked to the master reference library to prevent indexing shifts during final journal layout proofing.

We thank the reviewer for bringing this to our attention. The citations associated with Table 1 have been carefully reviewed and verified against the reference list. All in-text citations have been updated where necessary to ensure consistency with the reference library and to prevent numbering inconsistencies during the production process.
