# Supplementary material for: Early malaria risk screening in Nigerian minors using AutoML and cluster-based analysis of non-clinical survey data
Source: PLOS Digit Health. 2026 Sep 23;5(9):e0001736. doi: 10.1371/journal.pdig.0001736 (PMC13600517; doi:10.1371/journal.pdig.0001736)
Supplement: Supplementary file 2 [file pdig.0001736.s001.docx]

**S1 Table**. Final selected hyperparameter values by Optuna for all machine learning models trained on the 14-feature dataset, Cluster 1, and Cluster 2.

| **Dataset** | **Model** | **Hyperparameter** | **Optimal Value** |
| --- | --- | --- | --- |
| **14-Feature Dataset** | **GLM (H2O AutoML)** | Family / Link | Binomial / Logit |
|  |  | Solver | Coordinate Descent |
|  |  | Cross-Validation Folds (nfolds) | 10 |
|  |  | Alpha (α) / Lambda Search | 0.0 / True (nlambdas = 30) |
|  |  | Standardization / Missing Values | True / Mean Imputation |
|  | **SVC** | Kernel / Gamma Type | RBF / Auto |
|  |  | Regularization Parameter (C) | 1.7621 |
|  |  | Tolerance (tol) / Shrinking | 1.3002 × 10⁻⁴ / True |
|  | **Logistic Regression (LR)** | Penalty / Solver | L2 / LBFGS |
|  |  | Regularization Parameter (C) | 3.6535 |
|  |  | Max Iterations / Fit Intercept | 3517 / False |
|  | **XGBoost (XGB)** | Estimators (n_estimators) / Learning Rate | 1602 / 0.0803 |
|  |  | Max Depth / Min Child Weight | 4 / 15 |
|  |  | Subsample / Colsample (bytree) | 0.1715 / 0.1894 |
|  |  | Reg Alpha (α) / Reg Lambda (λ) | 1.1409 / 4.4925 |
|  |  | Scale Pos Weight / Gamma | 1.1204 / 0.0088 |
|  | **Decision Tree (DT)** | Criterion / Splitter | Gini / Best |
|  |  | Max Depth / Max Leaf Nodes | 29 / 163 |
|  |  | Min Samples Split / Min Samples Leaf | 38 / 32 |
|  |  | Max Features / Class Weight | sqrt / Balanced |
| **Cluster 1** | **GLM (H2O AutoML)** | Family / Link / Solver | Binomial / Logit / Coordinate Descent |
|  |  | Cross-Validation Folds / Alpha (α) | 10 / 0.0 |
|  | **SVC** | Kernel / Gamma Type / Shrinking | RBF / Auto / True |
|  |  | Regularization Parameter (C) / Tolerance | 0.0152 / 0.0671 |
|  | **Logistic Regression (LR)** | Penalty / Solver / Intercept | None / SAGA / False |
|  |  | Regularization Parameter (C) / Max Iter | 3.6822 / 884 |
|  | **XGBoost (XGB)** | Estimators / Learning Rate / Depth | 958 / 0.0644 / 19 |
|  |  | Min Child Weight / Subsample | 14 / 0.3633 |
|  |  | Reg Alpha (α) / Reg Lambda (λ) | 0.0508 / 6.4660 |
|  |  | Scale Pos Weight / Gamma | 1.3192 / 4.4552 |
|  | **Decision Tree (DT)** | Criterion / Splitter | Entropy / Random |
|  |  | Max Depth / Max Leaf Nodes | 32 / 25 |
|  |  | Min Samples Split / Min Samples Leaf | 27 / 19 |
| **Cluster 2** | **GLM (H2O AutoML)** | Family / Link / Solver | Binomial / Logit / Coordinate Descent |
|  |  | Cross-Validation Folds / Alpha (α) | 10 / 0.0 |
|  | **SVC** | Kernel / Gamma Type / Shrinking | RBF / Scale / True |
|  |  | Regularization Parameter (C) / Tolerance | 4.6477 × 10⁻⁴ / 0.0017 |
|  | **Logistic Regression (LR)** | Penalty / Solver / Intercept | L2 / Newton-Cholesky / True |
|  |  | Regularization Parameter (C) / Max Iter | 9.7171 × 10⁻⁴ / 199 |
|  | **XGBoost (XGB)** | Estimators / Learning Rate / Depth | 935 / 0.0138 / 7 |
|  |  | Min Child Weight / Subsample | 4 / 0.3423 |
|  |  | Reg Alpha (α) / Reg Lambda (λ) | 2.7337 × 10⁻⁴ / 0.0463 |
|  |  | Scale Pos Weight / Gamma | 1.5596 / 0.1577 |
|  | **Decision Tree (DT)** | Criterion / Splitter | Entropy / Best |
|  |  | Max Depth / Max Leaf Nodes | 15 / 149 |
|  |  | Min Samples Split / Min Samples Leaf | 4 / 34 |
