# Supplementary material for: Early malaria risk screening in Nigerian minors using AutoML and cluster-based analysis of non-clinical survey data
Source: PLOS Digit Health. 2026 Sep 23;5(9):e0001736. doi: 10.1371/journal.pdig.0001736 (PMC13600517; doi:10.1371/journal.pdig.0001736)
Supplement: Peer review history

# Peer review: Decision Letter 0

**DOI:** 10.1371/journal.pdig.0001736.r001

**Authors:** Henry Horng-Shing Lu, Cleva Villanueva

8 Jun 2026

Response to Reviewers Revised Manuscript with Track Changes Manuscript **Journal Requirements:**

1. Please ensure that your Ethics Statement is available in its entirety at the beginning of your Methods section, under a subheading 'Ethics Statement'.

2. We ask that a manuscript source file is provided at Revision. Please upload your manuscript file as a .doc, .docx, .rtf or .tex.

3. Please provide separate figure files in .tif or .eps format.

For more information about figure files please see our guidelines:  LINK

https://journals.plos.org/digitalhealth/s/figures

https://journals.plos.org/digitalhealth/s/figures#loc-file-requirements

4. We have noticed that you have uploaded Supporting Information files, but you have not included a list of legends. Please add a full list of legends for your Supporting Information files after the references list.

5. In the online submission form, you indicated that "The dataset utilized in this research is openly accessible through the Nigeria Demographic and Health Survey. Authorization to use the data is provided by the DHS Program and can be obtained through their official website at https://dhsprogram.com/Methodology/Survey-Types/DHS.cfm. The authors do not have the right to distribute or share the dataset.".

All PLOS journals now require all data underlying the findings described in their manuscript to be freely available to other researchers, either

1. In a public repository,

2. Within the manuscript itself, or

3. Uploaded as supplementary information.

This policy applies to all data except where public deposition would breach compliance with the protocol approved by your research ethics board. If your data cannot be made publicly available for ethical or legal reasons (e.g., public availability would compromise patient privacy), please explain your reasons by return email and your exemption request will be escalated to the editor for approval. Your exemption request will be handled independently and will not hold up the peer review process, but will need to be resolved should your manuscript be accepted for publication. One of the Editorial team will then be in touch if there are any issues.

**Additional Editor Comments (if provided):**

The study addresses an important public health challenge and explores the use of machine learning approaches for malaria risk screening using nationally representative survey data. The manuscript has several strengths, including the use of a large dataset, comparison of multiple machine learning methods, the application of clustering techniques to identify subpopulations, and the focus on an important pediatric population in a malaria-endemic setting.

After careful consideration of the manuscript and the reviewers’ comments, I believe that substantial revisions are required before the manuscript can be considered for publication. The principal issues that should be addressed are summarized below.

Major Issues

1. Potential Target Leakage and Consistency with the Study Objective

The manuscript presents the proposed framework as a non-clinical malaria screening approach based on socio-demographic and household characteristics. However, some of the most important predictors included in the models appear to be clinical or laboratory-derived variables, including malaria rapid diagnostic test results and Plasmodium falciparum status.

The authors should carefully reconsider the inclusion of these variables, provide a strong justification for their use, and discuss their implications for the intended use of the model. Ideally, the authors should evaluate model performance after removing clinical and laboratory variables to determine whether the framework can truly function as a non-clinical screening tool.

2. Survey Design and Representativeness

Additional information is needed regarding the handling of the Nigeria Malaria Indicator Survey design. It is currently unclear whether sampling weights, clustering, and stratification were incorporated appropriately into the analyses.

Given the complex survey design of the MIS dataset, the authors should provide a detailed explanation of how these elements were handled and discuss any implications for the validity and generalizability of the findings.

3. Missing Figures and Reproducibility

Several figures referenced in the manuscript were not available for review. These include confusion matrices, ROC curves, feature importance plots, clustering visualizations, and silhouette analyses.

These figures are essential for evaluating model performance, class separation, clustering validity, and interpretability. The authors should ensure that all referenced figures are included in the revised submission.

4. Clinical Translation and Practical Implementation

The manuscript would benefit from a more detailed discussion regarding how the proposed framework could be implemented in real-world settings.

Specifically, the authors should clarify:

How community health workers or healthcare providers would use the model in practice.

How the required variables would be collected at the point of care.

How missing information would be handled.

Whether a digital implementation strategy is envisioned.

This discussion is particularly important given that many predictor variables represent household-level or structural characteristics rather than individual clinical observations.

5. External Validation

The lack of external validation should be discussed more explicitly as an important limitation. Future validation using independent datasets would be necessary before deployment in clinical or public health settings.

Additional Methodological Clarifications

The revised manuscript should also address the following points:

Explain the rationale for removing variables with missing data and provide details regarding the number and type of excluded variables.

Clarify assumptions regarding missingness and justify the selected missing-data strategy.

Describe the clustering procedure in greater detail, including distance metrics and linkage criteria.

Provide greater transparency regarding the H2O AutoML configuration and imbalance-handling procedures.

Explain more clearly the characteristics that distinguish Clusters 1, 2, and 3.

Verify all reported performance metrics and tables for accuracy and consistency.

Discussion and Context

The discussion could be strengthened by:

Comparing the findings with additional relevant studies.

Providing a more balanced interpretation of the role of artificial intelligence and machine learning.

Reducing overly detailed descriptions of AI algorithms and focusing more on the practical implications of the findings.

**Reviewers' Comments:**

**Comments to the Author**

1. Does this manuscript meet PLOS Digital Health’s publication criteria?

Reviewer #1: Partly

Reviewer #2: Yes

Reviewer #3: Partly

**********

2. Has the statistical analysis been performed appropriately and rigorously?

Reviewer #1: I don't know

Reviewer #2: I don't know

Reviewer #3: No

**********

3. Have the authors made all data underlying the findings in their manuscript fully available (please refer to the Data Availability Statement at the start of the manuscript PDF file)?

The PLOS Data policy

Reviewer #1: Yes

Reviewer #2: Yes

Reviewer #3: Yes

**********

4. Is the manuscript presented in an intelligible fashion and written in standard English?

Reviewer #1: Yes

Reviewer #2: Yes

Reviewer #3: Yes

**********

Reviewer #1: The handling of Nigeria MIS survey design seems a bit insufficient because it gave me an impression that all observations were treated as independent and unweighted.....................................

Reviewer #2: Thank you for the opportunity to review the manuscript entitled “Early Malaria Risk Screening in Nigerian Minors Using AutoML and Cluster-Based Analysis of Non-Clinical Survey Data".

The authors of this manuscript introduced an AutoML-driven framework for non-clinical early malaria risk screening, using household and socio-economic data from the nationally representative Nigeria Malaria Indicator Survey (MIS) 2021. They used 15 significant predictors to develop XGBoost, SVC, LR, Decision Tree, LR, and H2O AutoML. Then, the top ten important features were selected, AHC was applied, and 3 clusters were introduced. After cluster-based model training, it was shown that H2O AutoML GRL had the best F2 score in most of the clusters and when the clusters were aggregated.

The manuscript described a clinically important issue and had comprehensive methodology, proper use of the F2 score, and spatial analysis. However, several methodological concerns should be addressed before the manuscript can be considered for publication. Additionally, the data were not fully available; however, the authors provided an explanation in the Data Availability Statement.

1. The authors may wish to mention other relevant studies in the discussion part.

2. Authors should mention why all partially missing features were removed. How many features were removed, and which specific features were excluded? Were they clinically or epidemiologically important?

3. Including positive RDTs as a feature can lead to potential leakage. If the objective is to develop an early screening framework based primarily on household and socio-demographic factors, the authors should consider excluding RDT-related variables from the predictive modelling pipeline or provide stronger justification for their inclusion.

4. Authors may wish to clearly mention external validation in the limitation part.

5. Cluster interpretation should be mentioned clearly. What distinguishes clusters 1, 2, and 3?

6. Some tables appear to contain identical performance values across different models. Please ensure they are reported correctly.

7. Some parts of the manuscript overly discussed AI models. These explanations may be unnecessarily detailed for the target readership and could be shortened to improve readability and maintain focus on the study’s methodological contribution and findings.

Reviewer #3: Major Revisions Required:

1. Target Contamination and Data Leakage (The 15-Feature Model)

The manuscript frames this work as a "non-clinical early malaria risk screening framework leveraging household and socio-economic data" to step in when rapid diagnostic tests (RDTs) or microscopy are delayed or unavailable. However, the 15-feature model includes "Presence of species: falciparum (Pf)" and "Result of malaria rapid test" as heavily weighted baseline features.

- "Presence of species: falciparum" requires laboratory microscopy or PCR to be determined. If a clinician already possesses parasitological laboratory confirmation of the species, a non-clinical machine learning screening model is redundant.

- Incorporating the "Result of malaria rapid test" to predict the final blood smear means the model is using a clinical diagnostic proxy of the target. This directly contradicts the premise that the tool operates when clinical testing is unavailable.

- Action Required: The authors must strictly isolate the non-clinical boundary. All clinical/laboratory variables must be completely removed from both the national and cluster-specific screening models. The authors must retrain the pipelines purely on true socio-demographic, environmental, and household features, and transparently discuss the resulting shifts in performance metrics.

2. Missing Evaluation Figures

The main text frequently references essential visual aids that are entirely absent from the provided manuscript files:

- Page 25 references Figures 3 through 7 (Confusion Matrices) and Figure 8 (AUC-ROC Curves).

- Page 25 references Figure 9 (Variable Importance of Gradient Boosting).

- Page 28 references Figure 10 (2D Projection via MCA) and Figure 11 (Silhouette Profile).

- Action Required: The authors must embed or provide these figures. Reviewers cannot validate classification boundaries, class-imbalance impacts, or clustering thresholds without inspecting these plots.

3. Translational and Operational Feasibility

The discussion section should address how an macro-level national household survey model translates to an active point-of-care digital health intervention. Features such as "Wealth index combined", "Type of toilet facility", and "Time to get to water source" are structural, seasonal, or aggregate indicators.

- Action Required: Clarify how a community health worker in a low-resource setting would operationalize this score during an acute clinical presentation. Is there an intended mobile interface? How will missing data fields at the individual level be dynamically accommodated by the H2O Stacked Ensemble?

Minor Revisions and Technical Corrections:

4. Clustering Reproducibility

The authors state that an optimal Silhouette Score of 0.510 was achieved with three clusters using Agglomerative Hierarchical Clustering. To ensure complete technical reproducibility, please explicitly report the distance metric (e.g., Euclidean) and the linkage criteria (e.g., Ward’s, Complete) utilized.

5. Hyperparameter Transparency in AutoML Class Imbalance

The manuscript notes that traditional ML models were under-sampled to handle class imbalance, while H2O AutoML was left untouched because it "handles imbalanced data internally." By default, tree-based algorithms inside H2O maximize standard accuracy. Please state the exact parameters passed to the H2O pipeline (e.g., balance_classes=True, or explicit setting of stopping_metric="F2") to clarify how the pipeline prioritized recall over majority-class dominance.

6. Citation Mismatch in Table 1

In Section 1 (Table 1), recent literature benchmarks are hardcoded as static text brackets (e.g., [16], [17]). Ensure these entries are properly linked to the master reference library to prevent indexing shifts during final journal layout proofing.

**********

what does this mean?). If published, this will include your full peer review and any attached files.

**Do you want your identity to be public for this peer review?**  If you choose “no”, your identity will remain anonymous but your review may still be made public.

For information about this choice, including consent withdrawal, please see our Privacy Policy

Reviewer #1: No

Reviewer #2: No

Reviewer #3: No

**********

**Figure resubmission:**

**Reproducibility:**

To enhance the reproducibility of your results, we recommend that authors of applicable studies deposit laboratory protocols in protocols.io, where a protocol can be assigned its own identifier (DOI) such that it can be cited independently in the future. Additionally, PLOS ONE offers an option to publish peer-reviewed clinical study protocols. Read more information on sharing protocols at https://plos.org/protocols?utm_medium=editorial-email&utm_source=authorletters&utm_campaign=protocols

# Author comment: Author response to Decision Letter 1

**DOI:** 10.1371/journal.pdig.0001736.r002

7 Aug 2026

Attachment Submitted filename: Response to Reviewers.pdf

# Peer review: Decision Letter 1

**DOI:** 10.1371/journal.pdig.0001736.r003

**Authors:** Henry Horng-Shing Lu, Cleva Villanueva

1 Sep 2026

Early Malaria Risk Screening in Nigerian Minors Using AutoML and Cluster-Based Analysis of Non-Clinical Survey Data

PDIG-D-26-00617R1

Dear Dr. Nafisa Mahbub,

We are pleased to inform you that your manuscript 'Early Malaria Risk Screening in Nigerian Minors Using AutoML and Cluster-Based Analysis of Non-Clinical Survey Data' has been provisionally accepted for publication in PLOS Digital Health.

Before your manuscript can be formally accepted you will need to complete some formatting changes, which you will receive in a follow-up email from a member of our team.

Please note that your manuscript will not be scheduled for publication until you have made the required changes, so a swift response is appreciated.

IMPORTANT: The editorial review process is now complete. PLOS will only permit corrections to spelling, formatting or significant scientific errors from this point onwards. Requests for major changes, or any which affect the scientific understanding of your work, will cause delays to the publication date of your manuscript.

If your institution or institutions have a press office, please notify them about your upcoming paper to help maximize its impact. If they'll be preparing press materials, please inform our press team as soon as possible -- no later than 48 hours after receiving the formal acceptance. Your manuscript will remain under strict press embargo until 2 pm Eastern Time on the date of publication. For more information, please contact digitalhealth@plos.org.

Thank you again for supporting Open Access publishing; we are looking forward to publishing your work in PLOS Digital Health.

Best regards,

Cleva Villanueva, M.D., Ph.D.

Academic Editor

PLOS Digital Health

***********************************************************

**Additional Editor Comments (if provided):**

The revised manuscript, together with your responses to the reviewers’ comments, has been carefully evaluated. The revisions have adequately addressed the concerns and recommendations raised during the previous round of peer review, and the manuscript has been improved accordingly.

In its current form, the manuscript meets the requirements for publication in PLOS Digital Health. I am therefore pleased to recommend acceptance of the manuscript for publication

**Reviewer Comments (if any, and for reference):**

Reviewer's Responses to Questions

**Comments to the Author**

Reviewer #2: All comments have been addressed

**********

publication criteria?

Reviewer #2: Yes

**********

3. Has the statistical analysis been performed appropriately and rigorously?

Reviewer #2: I don't know

**********

4. Have the authors made all data underlying the findings in their manuscript fully available (please refer to the Data Availability Statement at the start of the manuscript PDF file)?

The PLOS Data policy

Reviewer #2: Yes

**********

5. Is the manuscript presented in an intelligible fashion and written in standard English?

Reviewer #2: Yes

**********

Reviewer #2: I have reviewed the revised manuscript and the authors’ responses to my previous comments. The authors have adequately addressed the concerns raised during the previous review, and the manuscript has been substantially improved. In particular, the revisions to the predictive modelling framework and the removal of variables associated with potential target leakage have strengthened the methodological rigor of the study. The authors have also discussed external validation and the practical implementation of the proposed framework. Additionally, the feature selection procedure and the interpretation of the clustering results have been clarified.

The responses are clear, and the corresponding revisions have been appropriately incorporated into the manuscript. I have no further major concerns regarding the revised version. I therefore recommend acceptance of the manuscript for publication.

**********

what does this mean?). If published, this will include your full peer review and any attached files.

**Do you want your identity to be public for this peer review?**  If you choose “no”, your identity will remain anonymous but your review may still be made public.

For information about this choice, including consent withdrawal, please see our Privacy Policy

Reviewer #2: No

**********
